# Mechanistic basis of substrate–O_2_ coupling within a chitin-active lytic polysaccharide monooxygenase: An integrated NMR/EPR study

**DOI:** 10.1073/pnas.2004277117

**Published:** 2020-07-28

**Authors:** Gaston Courtade, Luisa Ciano, Alessandro Paradisi, Peter J. Lindley, Zarah Forsberg, Morten Sørlie, Reinhard Wimmer, Gideon J. Davies, Vincent G. H. Eijsink, Paul H. Walton, Finn L. Aachmann

**Affiliations:** ^a^Norwegian Biopolymer Laboratory (NOBIPOL), Department of Biotechnology and Food Science, Norwegian University of Science and Technology, N-7491 Trondheim, Norway;; ^b^Department of Chemistry, University of York, Heslington, York YO10 5DD, United Kingdom;; ^c^School of Chemistry, University of Manchester, Manchester M13 9PL, United Kingdom;; ^d^Photon Science Institute, University of Manchester, Manchester M13 9PL, United Kingdom;; ^e^Faculty of Chemistry, Biotechnology and Food Science, Norwegian University of Life Sciences, N-1432 Ås, Norway;; ^f^Department of Chemistry and Bioscience, Aalborg University, 9220 Aalborg Ø, Denmark

**Keywords:** lytic polysaccharide monooxygenase, copper, chitin, EPR, NMR

## Abstract

Lytic polysaccharide monooxygenases (LPMOs) have a unique ability to activate molecular oxygen for subsequent oxidative cleavage of glycosidic bonds. To provide insight into the mode of action of these industrially important enzymes, we have performed an integrated NMR/electron paramagnetic resonance (EPR) study into the detailed aspects of an AA10 LPMO–substrate interaction. Using NMR spectroscopy, we have elucidated the solution-phase structure of *apo*-*Bl*LPMO10A from *Bacillus licheniformis*, along with solution-phase structural characterization of the Cu(I)-LPMO, showing that the presence of the metal has minimal effects on the overall protein structure. We have, moreover, used paramagnetic relaxation enhancement (PRE) to characterize Cu(II)-LPMO by NMR spectroscopy. In addition, a multifrequency continuous-wave (CW)-EPR and ^15^N-HYSCORE spectroscopy study on the uniformly isotope-labeled ^63^Cu(II)-bound ^15^N-*Bl*LPMO10A along with its natural abundance isotopologue determined copper spin-Hamiltonian parameters for LPMOs to markedly improved accuracy. The data demonstrate that large changes in the Cu(II) spin-Hamiltonian parameters are induced upon binding of the substrate. These changes arise from a rearrangement of the copper coordination sphere from a five-coordinate distorted square pyramid to one which is four-coordinate near-square planar. There is also a small reduction in metal–ligand covalency and an attendant increase in the d(x^2^−y^2^) character/energy of the singly occupied molecular orbital (SOMO), which we propose from density functional theory (DFT) calculations predisposes the copper active site for the formation of a stable Cu–O_2_ intermediate. This switch in orbital character upon addition of chitin provides a basis for understanding the coupling of substrate binding with O_2_ activation in chitin-active AA10 LPMOs.

## Significance

Lytic polysaccharide monooxygenases (LPMOs) have unique catalytic centers, at which a single copper catalyzes the oxidative cleavage of a glycosidic bond. The mechanism by which LPMOs activate molecular oxygen is key to understanding copper (bio)catalysis but remains poorly understood, largely because the insoluble and heterogeneous nature of LPMO substrates precludes the use of usual laboratory techniques. Using an integrated NMR/EPR approach, we have unraveled structural and electronic details of the interactions of an LPMO from *Bacillus licheniformis* and β-chitin. EPR spectroscopy on uniformly isotope ^15^N-labeled ^63^Cu(II)-LPMO provided insight into substrate-driven rearrangement of the copper coordination sphere that predisposes the enzyme for O_2_ activation.

The sustainable use of polysaccharides from lignocellulosic biomass as a feedstock in the production of biofuels and biomaterials is key to reducing dependency on fossil fuels. In this regard, chitin, an abundant insoluble polysaccharide found in the exoskeletons of arthropods and the cell walls of fungi, has often been proposed as a potential feedstock for conversion into high-value biomaterials (1–3). Given this impetus, the efficient processing of chitin through enzymatic breakdown into its constituent sugars is an attractive means of realizing its full chemical and calorific potential, and indeed that of other polysaccharides such as cellulose. It is unsurprising, therefore, that the commercial use of enzyme mixtures for this purpose is widespread, where the content of these mixtures includes a range of glycoside hydrolases (GHs) and, more recently, copper-dependent redox enzymes known as lytic polysaccharide monooxygenases (LPMOs).

LPMOs are currently classified as auxiliary activity (AA) families 9–11 and 13–16 in the CAZy database (4, 5). They have been shown to augment dramatically the activity of GHs, probably by reducing the crystallinity of their substrates (6–12), and now, alongside GH enzymes, are seen as key components in the efficient processing of abundant biomass. Accordingly, there is much interest in increasing the efficiency of LPMOs through a deeper understanding of their molecular and electronic features. From previous studies, it is known that LPMOs have an oxidative mode of action on their substrates. This oxidation proceeds through hydrogen-atom abstraction from either the C1 or C4 carbon in 1–4 linked polysaccharides such as cellulose or chitin (8, 9, 13, 14) to generate the respective hydroxylated product, from which elimination leads to cleavage of the glycosidic bond. The mechanism likely involves the formation of a copper-bound reactive oxygen species that arises from the reaction of the copper active site of the LPMO with O_2_ and a reducing agent, or with hydrogen peroxide (9, 14–18).

In the context of the O_2_ mechanism, there is consensus regarding the initial step of the catalytic cycle in which Cu(I)-LPMO reacts with O_2_ to give a Cu(II) and superoxide (14, 19). Any Cu(II)–superoxide complex formed in this manner may then oxidize the substrate or, with the addition of further electrons and protons, go on to form “high valent” copper–oxygen intermediates, which have not yet been observed experimentally in LPMOs. Proposals for these intermediates, however, have come from computational studies and include Cu(II)-oxyl and Cu(III)-hydroxide (19–25). An equivalent outcome is achieved by the direct reaction of Cu(I)-LPMO with H_2_O_2_. Indeed, density functional theory (DFT) calculations on AA9 LPMOs have shown that the O_2_ and H_2_O_2_ reaction pathways converge on a common intermediate (26, 27). Recent work on the mechanism of H_2_O_2_-driven LPMO reactions has led to suggestions that hydroxyl radicals could also play a role (15, 26, 28). If such species are the oxidants or, indeed part of an overall catalytic cycle, then the role of the substrate is essential (15). For instance, in the presence of substrate, it has been shown by DFT calculations for both AA9 LPMOs (26) and, more recently AA10 LPMOs (28), that any hydroxyl generated in this manner is redirected by active-site residues back toward the copper ion. This redirection forms a protein-bound Cu(II)-oxyl species, which then acts as the key oxidizing intermediate in the catalytic cycle, thus avoiding the deleterious oxidative effects of the hydroxyl intermediate.

In this context, a major question facing the LPMO world is what mechanisms are employed by the enzymes to couple the presence of substrate to the generation of oxidizing intermediates, thus avoiding the deleterious oxidation of the protein? Following initial proposals on the mechanism of LPMOs (8, 19) and the importance of considering the substrate’s role, a growing number of studies are indeed now showing that substrate binding and LPMO catalysis are coupled (18, 22, 29–32). For instance, both Borisova et al. (29) and Frandsen et al. (22) demonstrated that the spin-Hamiltonian parameters of the Cu(II) in some AA9 LPMOs shift significantly upon the LPMO binding to the substrate, potentially indicating a “trigger” mechanism in which the substrate regulates the catalytic mechanism and protects from inactivation pathways (33). For family AA10 LPMOs, the dissociation constants (*K*_d_) of copper binding have been determined to be 6 to 55 nM for Cu(II) and ∼1 nM for Cu(I) (34, 35). In this respect, Kracher et al. (30) linked the oxidation state of the copper to binding affinity for the substrate. More recently, based on modeling studies, Bissaro et al. (32) showed that chitin binding to *Sm*LPMO10A results in a constrained copper site geometry that includes a tunnel through which small cosubstrates could diffuse in the presence of substrate. It has also been shown that the presence of substrate enhances the stability of LPMOs (15, 33, 36, 37). Evidently, whether an LPMO is bound to its polysaccharide substrate not only affects the stability of the LPMO, but it also likely determines the mechanism(s) by which the oxidative intermediates are generated (19).

In order to understand better the mode of action of LPMOs, with particular focus on the role of copper, we describe herein an integrated NMR/EPR spectroscopy approach designed to investigate the effect of copper- and substrate-binding to a chitin-active LPMO from *Bacillus licheniformis* (hereinafter called *Bl*LPMO10A) and in particular the enzyme’s ability to activate O_2_ at the copper center. It is the first study of its kind on LPMOs, in which we have taken advantage of the ^15^N-labeling that is required for the NMR study to simplify and constrain the analysis of the spin-Hamiltonian parameters obtainable from EPR spectroscopy. Using this approach, we solved the NMR structure of *apo*-*Bl*LPMO10A and Cu(I)- *Bl*LPMO10A, assessed dynamic features derived from relaxation data (*T*_1_, *T*_2_, and {^1^H}-^15^N NOE), and evaluated the structural effects of Cu(I) and Cu(II) binding. In tandem, EPR spectroscopy performed on both natural isotopic abundance samples of ^63^Cu(II)-*Bl*LPMO10A and on uniformly isotope-labeled ^63^Cu(II)-^15^N-*Bl*LPMO10A has allowed the determination of hyperfine couplings. These couplings provide insights into the rearrangement of the copper coordination sphere upon substrate binding at a high level of detail, leading to the proposal of a potential substrate–O_2_ coupling mechanism in chitin-active AA10 LPMOs. The work further sets the scene for future integrated NMR/EPR studies and in-depth EPR investigation of solid-state samples, i.e., investigations performed with the protein bound to its natural solid state substrate—an important aspect of all LPMO–substrate studies.

## Results and Discussion

### Functional Characterization of *Bl*LPMO10A.

*Bl*LPMO10A was recombinantly produced in *Escherichia coli* and copper saturated using previously established methods (38, 39). Activity assays in reactions with 2 mM ascorbic acid as reductant showed that the enzyme is active on β-chitin and also has low activity on α-chitin (*SI Appendix*, Fig. S1*A*). In reactions without added reductants, product formation was not observed. The profile of oxidized oligomers (*SI Appendix*, Fig. S1*A*) showed a dominance of products with an even number of sugar units, which is a feature that is typical for LPMOs acting on crystalline chitin (9, 40, 41). As expected, binding to β-chitin was observed (*SI Appendix*, Fig. S1*B*).

### *apo*- and Cu(I) Structures of *Bl*LPMO10A.

**Fig. 1.**
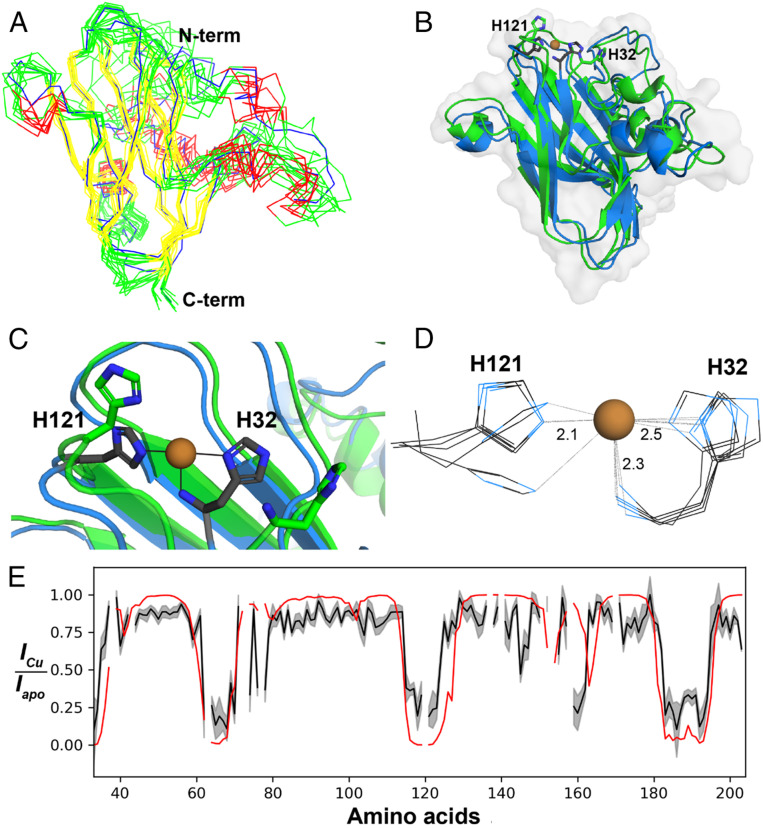
Structures of *apo*- and Cu(I)-*Bl*LPMO10A. (*A*) Ensemble of the 10 lowest-energy conformers of *apo*-*Bl*LPMO10A (PDB ID code 5LW4) in stereo representation. Helices are colored red, loops are colored green, and strands are colored yellow; the lowest CYANA target energy conformer is colored blue. The overall backbone rmsd of the ensemble is 2.41 Å, while the rmsd of the regions containing α-helices (residues 41–47, 57–61, 82–84, 89–94, and 159–162) and β-sheets (residues 33–36, 37–40, 103–107, 110–117, 125–132, 150–154, 164–168, 175–184, 185–190, and 191–201) is 1.48 Å. (*B*) Overlay of *apo*-*Bl*LPMO10A (green) and Cu(I)-*Bl*LPMO10A (PDB ID code 6TWE; blue). The copper atom is shown as an orange sphere, and the side chains of His32 and His121 are shown as sticks. The backbone (C^α^, N, C′) rmsd between the *apo* ensemble and the Cu(I) ensemble is 0.9 Å. (*C*) Zoomed-in view of the overlay in *B* showing details of the copper site. (*D*) Ensemble of five lowest-energy conformers of Cu(I)-*Bl*LPMO10A, showing the copper site. Average distances from each N atom to the Cu atom are indicated. (*E*) PRE effects upon adding Cu(II) to *apo*-*Bl*LPMO10A. The black line shows the normalized H^N^, N signal intensity upon addition of Cu(II) to ^13^C- and ^15^N-labeled *apo*-*Bl*LPMO10A in a 1:2 ratio, relative to the intensity for the *apo*-enzyme, with errors shown in gray. The red line shows PREs calculated using the Cu(I)-*Bl*LPMO10A ensemble. Gaps in the data represent missing assignments for amino acid residues (e.g., Pro).

The solution structure of *apo*-*Bl*LPMO10A (Protein Data Bank [PDB] ID code 5LW4) (Fig. 1*A*) was elucidated using NMR spectroscopy. The structure was calculated in CYANA using 1,623 nuclear Overhauser effect (NOE)-derived distance constraints, 264 TALOS-N determined torsion angle constraints, and one disulfide bridge (Cys45–Cys56) constraint (*SI Appendix*, Table S1). Like all other known LPMOs, *Bl*LPMO10A has the typical fibronectin type III-like β-sandwich fold, in this case composed of eight β-strands that form a three-stranded and a five-stranded β-sheet, connected through loops of various lengths. The three-stranded sheet is composed solely of antiparallel strands, whereas the five-stranded sheet contains four antiparallel strands and one short parallel strand. The stretch of 66 amino acids that connects the first (Phe35–Lys38) and the second (His105–Met107) β-strands is composed of irregular loop regions, two α-helices and one 3_10_-helix. A third short α-helix occurs in the loop between the fifth (Phe146–Pro154) and the sixth (Gly176–Val185) β-strands.

NMR investigations of LPMOs are usually carried out using the *apo*-proteins, in order to avoid the detrimental signal reduction caused by the paramagnetic relaxation enhancement (PRE) effect brought about by the nature of the type II copper site (42). Here, we exploited the PRE effect to gain insights into the effects of Cu(II) on the structure of *apo*-*Bl*LPMO10A (*SI Appendix*, Fig. S2). Cu(II) was added to a sample of *apo*-*Bl*LPMO10A and the PRE effect was evaluated by comparing signal intensity reduction in ^15^N-heteronuclear single quantum coherence (HSQC) spectra with PREs calculated using Cu(I)-*Bl*LPMO10A structures, the *g*_iso_ values from Table 1, and relaxation parameters (*SI Appendix*, Fig. S3) (43). Residues nearest the copper coordination site showed more than 80% reduction in signal intensity (Fig. 1*E*). As expected, residues with the highest signal intensity reduction are located within a 12-Å radius from the Cu(II) coordination site (*SI Appendix*, Fig. S2). An exception is a short helix (Ala160–Arg162; *SI Appendix*, Fig. S3) that is further away than the expected 12 Å. This deviation could indicate structural differences beyond the copper-active site between copper-bound and *apo*- forms.

**Table 1.** Spin-Hamiltonian parameters for ^63^Cu(II)-*Bl*LPMO10A and ^63^Cu(II)-^15^N-*Bl*LPMO10A at pH 5.5 with and without squid pen β-chitin

		^63^Cu*-Bl*LPMO10A	^63^Cu*-*^15^N*-Bl*LPMO10A		^63^Cu*-Bl*LPMO10A + β-chitin	^63^Cu*-*^15^N*-Bl*LPMO10A+ β-chitin	
		X-band	X-band	Q-band	X-band	X-band	Q-band
*g* values	*g*_*1*_	2.027	2.029	2.032	2.042	2.038	2.046
	*g*_*2*_	2.095	2.081	2.112	2.053	2.046	2.057
	*g*_*3*_	2.261	2.261	2.260	2.205	2.209	2.208
	*g*_iso_	2.128	2.124	2.135	2.101	2.098	2.104
A_Cu_, /MHz	|A_1_|	255	255	255	80	88	80
	|A_2_|	110	115	115	85	95	90
	|A_3_|	336	336	340	620	610	610
Calculated*	*A*_iso_	10	11	10	−208 or −262	−205 or −264	−207 or −260
SHF A_N_ principal values,^†^ /MHz		43, 43, 28	60, 60, 40	60, 60, 40	40, 40, 32	56, 56, 45	55, 55, 45
		±5	±5		±2	±2	
*g*_Cu_ strains		0, 0.02, 0.007	0, 0.025, 0.005	0, 0.045, 0.007	0.004, 0, 0.007	0.005, 0, 0.009	0.005, 0, 0.003
A_Cu_ strains, /MHz		130, 55, 160	160, 60, 160	20, 10, 120	20, 20, 20	10, 10, 10	90, 40, 240
Linewidths		0.6, 0.6	0.6, 0.6	4.5, 4.5	0.4, 0.4	0.5, 0.6	1.3, 1.3
							
Frequency, /GHz		9.3046	9.2973	35.00	9.2988	9.2884	35.05

For coupled nitrogen nuclei, only the principal coupling value could be determined from the simulations of the superhyperfine (SHF), which we presume is the coupling along the Cu–N bond; the three values in each spectrum refer to three different N nuclei, with the smallest value in each set being assigned to the NH_2_.

* Signs of A_1_ and A_2_ calculated from DFT (see main text).

^†^ Error estimated from quality of simulated fits.

Preparation of a Cu(I)-bound sample of ^13^C- and ^15^N-labeled *Bl*LPMO10A enabled structure determination of the reduced Cu(I)-LPMO in solution (PDB ID code 6TWE and Fig. 1). The structure was elucidated by using 1,209 distance restraints derived from nuclear Overhauser effect spectroscopy (NOESY) spectra of a Cu(I)-bound *Bl*LPMO10A sample together with force-field parameters for the copper site of another AA10, *Sm*LPMO10A (32). Overall, the Cu(I)-structure resembles the *apo*-structure (Fig. 1*B*), but there are clear differences in the internuclear ^1^H–^1^H distances at the copper site (Fig. 1*C*). The overall backbone (C^α^, N, C′) rmsd between the *apo*- and Cu(I)-structures is 0.9 Å. Analysis of NOESY spectra revealed that the presence of Cu(I) slightly affects internuclear distances between the β-strands, suggesting minor sliding movements in the β-strands upon copper binding. While Fig. 1*C* shows that copper binding has a strong effect on the conformation of the copper-coordinating His side chains, limitations inherent to NMR data preclude determination of exact atomic coordinates for the copper structure. These limitations are evident in Fig. 1*D*, which shows that the histidine side chains do not coordinate to the Cu with the expected T-shaped coordination geometry, which is chemically unreasonable. Fig. 1 *B* and *C* show that copper binding has a major effect on the conformation of the copper site. This conformational change is a result of the combined effects of introducing force-field parameters for the copper site and of structural changes encoded in NOE-derived distance restraints. Prior to this study, Cu(I)-structures of LPMOs had only been obtained by photoreduction of Cu(II) in the X-ray beam during data acquisition for crystallography or X-ray absorption studies (42).

### Heteronuclear Relaxation.

To gain insight into the motion of *Bl*LPMO10A in solution, {^1^H}-^15^N NOE, ^15^N-T_1_, and ^15^N-T_2_ were measured (*SI Appendix*, Fig. S3), and the rotational correlation time, τ_c_, which relates to molecular tumbling, was determined from the average T_1_/T_2_. The overall horizontal trends in *SI Appendix*, Fig. S3, suggest that the core of *Bl*LPMO10A is rigid, while loops, particularly in the first half of the protein, have reduced {^1^H}-^15^N NOE and increased T_2_ values that indicate some conformational flexibility. The rotational correlation time (τ_c_ = 10.2 ± 0.9 ns) was found to be similar to what would be expected for a globular protein of similar molecular weight [τ_c_ = 9.75 ± 0.46 ns for a 20-kDa globular protein (44)]. This finding indicates that *Bl*LPMO10A is a well-packed protein, as observed previously for other LPMOs.

### EPR Spectroscopy.

**Fig. 2.**
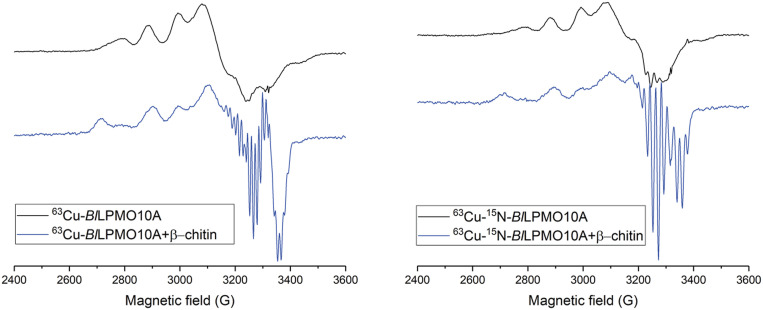
CW-EPR spectra of *Bl*LPMO10A. Spectra for ^63^Cu–*Bl*LPMO10A (*Left*) and ^63^Cu‒^15^N‒*Bl*LPMO10A (*Right*) before and after addition of squid pen β-chitin (black and blue lines, respectively). The spectra were recorded with 0.29 mM ^63^Cu–*Bl*LPMO10A and 0.17 mM ^63^Cu‒^15^N‒*Bl*LPMO10A, both in 20 mM MES buffer, pH 5.5, with 10% glycerol.

The availability of ^15^N-labeled enzyme offered the possibility of simultaneously determining the spin-Hamiltonian parameters of the Cu(II) unpaired electron for both ^14^N and ^15^N species, thereby providing a means of accurately determining their values. To this end, continuous-wave (CW)-EPR spectra were collected at both X-band and Q-band frequencies with pure ^63^Cu isotopes of LPMOs, from which a simultaneous fit of the spectra at both frequencies provided a doubly constrained and therefore reliable set of Cu(II) *g* values and hyperfine coupling constants (*A*_Cu_), along with nitrogen superhyperfine coupling constants. The data from these fits are presented in Table 1 and shown in Fig. 2 and *SI Appendix*, Fig. S4 (45).

In the absence of chitin, simultaneous fits of X- and Q-band spectra afforded a consistent set of copper spin-Hamiltonian parameters, characterized by rhombic principal *g* matrix values, a reduced ׀A_3_׀ value and large ׀A_1,2_׀ values [with respect to typical |A*|* values for axial Cu(II) systems], where the overall spectral envelope indicates a singly occupied molecular orbital (SOMO) with mostly d(x^2^−y^2^) character (Table 1). These values are similar to those obtained for other chitin-active AA10 LPMOs, but differ in that the ׀A_1_׀ value, which is more accurately determined in the present study through the use of two frequencies, is larger than previously reported (32, 35, 38), as is the corresponding *g*_1_ value.

**Fig. 3.**
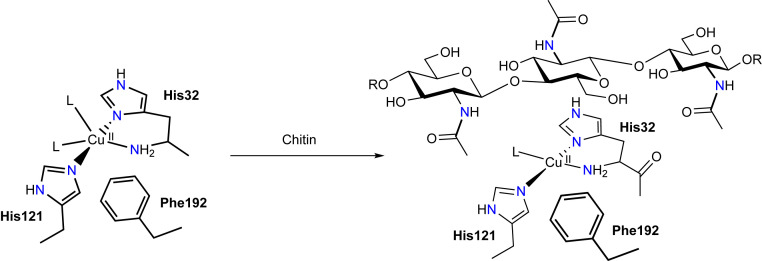
Schematic representation of the change in the coordination sphere of the copper ion upon binding of β-chitin (L = H_2_O or OH^−^).

The rhombic spin-Hamiltonian parameters, particularly the reduced *g*_3_ along with increased *g*_2_ (as compared to *g* values for typical axial Cu(II) systems), derive from d-orbital mixing which occurs in copper complexes that possess a distorted square-pyramidal coordination geometry (46). Indeed, such a coordination geometry in Cu(II)-*Bl*LPMO10A would be in accord with those previously observed in the crystal structures of AA10 LPMOs in the Cu(II) oxidation state (41, 47–49). In these structures, the base of the pyramid is defined by the three nitrogen atoms of the histidine brace and a water molecule. A further water molecule in the distal axial position, albeit slightly off axis with respect to the ideal geometry, completes the coordination sphere (Fig. 3, *Left*).

Addition of chitin flakes to the samples of Cu(II)-*Bl*LPMO10A led to significant changes in both X-band and Q-band EPR spectra (Fig. 2). Excellent simultaneous fits of the different frequency spectra could be obtained, with a notably high correlation in the areas in which superhyperfine coupling is evident (*SI Appendix*, Fig. S4). In the presence of chitin, the spectra are well defined with axial copper spin-Hamiltonian parameters (*g*_1_ ∼ *g*_2_ < *g*_3_) and a large ׀A_3_׀ value. The latter value leads to the appearance of an ostensible “overshoot” feature [also known as “extra absorption peak” (50)] at high field in the X-band spectrum, which has been suggested in other studies on LPMOs to arise from a low *g*_1_ value (2.018) (32). However, Q-band spectra of Cu(II)-*Bl*LPMO10A demonstrate that the high field feature in the X-band spectrum actually arises from the large ׀A_3_׀ value and that, when simulated with two frequencies, the perpendicular hyperfine constants are those typical for an axial Cu(II) complex in which there is little mixing into the d(x^2^−y^2^) SOMO from other metal-based orbitals and the *g* values are typical for an axial Cu(II) complex. Thus, in the presence of chitin, the copper coordination sphere is one that has near axial coordination symmetry, similar to that seen in most AA9 LPMO structures (Fig. 3, *Right*) where the equatorial plane of the copper is defined by the three nitrogen atoms of the histidine brace and the coordinating atom of an exogenous molecule (e.g., water, hydroxide), all held within a near-planar arrangement around the copper.

#### Substrate-induced effects at the active site.

While the NMR solution structure of Cu(I)-*Bl*LPMO10A offers qualitative insights into structural rearrangements induced by copper binding, it does not provide a high-resolution structure of the active site (Fig. 1*D*). It is thus not possible to corroborate the EPR parameters with DFT calculations based on the Cu(I)-*Bl*LPMO10A model of the active site. Therefore, we used ligand field theory enhanced by DFT (acronym DELFT for DFT-enhanced ligand field theory) to analyze the nature of the SOMO and its magnetic interactions (*SI Appendix*, *Supplementary Discussion*). In the DELFT method, DFT calculations over a range of functionals (*SI Appendix*, Table S3) are performed on the active site of a closely related AA10 LPMO (*Ba*AA10 from *Bacillus amyloliquefaciens*, 58% sequence identity to *Bl*LPMO10A) where the structure of the active site in its Cu(II) form is known from X-ray crystallography (41), and where *Ba*AA10 shows virtually identical CW-EPR spectra to those of *Bl*LPMO10A (35, 51), demonstrating that the two enzymes have a comparable arrangement at the active site and a similar response to the addition of chitin. On this basis, the coordinates for *Ba*AA10 were used as a basis for DFT calculations (*SI Appendix*, Figs. S7–S9). The objective of DELFT approach is not to obtain accurate spin-Hamiltonian parameters for the Cu and coordinating nitrogen atoms, not least because the calculation of such parameters with DFT is fraught with difficulty (52), but rather to determine the signs of the Cu hyperfine coupling constants, which cannot be ascertained experimentally from CW-EPR spectroscopy. Accordingly, it was found that, in the presence of substrate, A_2_ is negative and the sign of A_1_ is unclear, and that both A_1_ are A_2_ positive in the absence of substrate (*SI Appendix*, Tables S4–S7). Therefore, it is possible to calculate experimental *A*_iso_ values of approximately −205 or −265 MHz (depending on the sign of A_1_) in the presence of substrate and ∼10 MHz in its absence (Table 1), representing a significant shift in *A*_iso_ induced by the addition of substrate (*SI Appendix*, *Supplementary Discussion*).

In the context of ligand field theory, *A*_iso_ is a useful measure since it is determined, to second order within a fixed *g* matrix, by three main factors: 1) spin–orbit contributions, 2) the degree of Fermi contact of the unpaired electron with the copper nucleus, and 3) the spin density at the copper, which in turn can be related to the degree of metal–ligand covalency in the ground state. Which of these is the origin of the shift in *A*_iso_ value upon substrate binding in *Bl*LPMO10A can then be evaluated from the experimentally well-defined A_3_ hyperfine coupling value of the copper, using the following equation, which applies to the hyperfine coupling constants of a Cu(II) ion with d(x^2^−y^2^) SOMO in a distorted square pyramidal geometry:

$$A_{3}=-P_{\text{d}}\left[K+\frac{4\alpha_{GS}^{2}\left(a^{2}-b^{2}\right)}{7}-\frac{\left(3a-\sqrt{3}b\right)\Delta g_{2}}{14\left(a+\sqrt{3}b\right)}-\frac{\left(3a+\sqrt{3}b\right)\Delta g_{1}}{14\left(a-\sqrt{3}b\right)}-\Delta g_{3}\right].$$

In this equation, *P*_d_ = 1,180 cm^−1^, *a* = the orbital coefficient of the d(x^2^−y^2^) orbital, *b* = the orbital coefficient of the d(z^2^) orbital, $\alpha_{\text{GS}}^{2}$ = spin density on Cu, Δ*g*_n_ = *g*_n_ − 2.0023, and the Fermi contact −*P*_d_*K*, which can be calculated from the following:

$$A_{iso}=P_{\text{d}}\left[-K+\frac{1}{3}\left(\Delta g_{x}+\Delta g_{y}+\Delta g_{z}\right)\right].$$

Apart from the spin density, $\alpha_{GS}^{2}$, the only unknown in the first equation is the degree of d(z^2^) mixing into the ground state, denoted by the orbital coefficient *b*. [This value can be estimated from the difference in *g*_1_ and *g*_2_ values (53), giving a value of *b*^2^ to be ∼2%.] The values for the individual contributions to the *A*_iso_ can then be calculated (Table 2). Also shown in Table 2 are the equivalent theoretical values calculated using DFT of the active site of *Ba*AA10 in two forms, one with two exogenous water molecules coordinating to the copper (analogous to the active-site structure in the absence of substrate) and the other with a single water as the exogenous ligand, mimicking the substrate-bound state (see *SI Appendix*, Tables S3 and S4, and below for further discussion).

**Table 2.** Contributions to hyperfine coupling /MHz

Method	Fermicontact	Dipolarpara	Dipolarperp	Orbitalpara	Orbitalperp	Spin density
+ chitin,						
− no chitin						
	/MHz	/MHz	/MHz	/MHz	/MHz	α
DELFT −	−139	−533	281	332	70	0.82
DFT −	−178	−528	264	295	94	
						
DELFT +*	−324	−558	279	262	53	0.83
DELFT+^†^	−380	−504	252	264	50	0.75
DFT +	−348	−545	272	291	80	

Para, parallel direction; perp, perpendicular direction. +, presence of substrate; −, absence of substrate.

* Calculated with *A*_iso_ = −208 MHz.

^†^ Calculated with *A*_iso_ = −262 MHz.

This analysis shows that the DELFT and DFT approaches yield broadly consistent values and the same trends in how the contributors to hyperfine coupling constants change upon substrate addition, with the largest change appearing in the value of the Fermi contact. The DELFT approach also reveals that the spin density, $\alpha_{\text{GS}}^{2}$, in the presence of substrate takes one of two values (0.83 or 0.75) depending on the sign of *A*_1_ used in the calculation of *A*_iso_. It is likely that the value of 0.83 is the more reliable one, given that there are no significant changes in the identity of coordinating atoms to the copper upon the addition of substrate (i.e., we do not expect a large increase in the covalency of the copper–ligand bonds as would be required by a spin density value of 0.75). Thus, it appears as if the addition of substrate affords either no change or a slight decrease in the overall metal–ligand covalency.

Most contributors to the value of the hyperfine coupling are not altered much by the addition of substrate. However, the change in the value of Fermi contact parameter, −*P*_Cu_κ (*SI Appendix*, *Supplementary Discussion*), is significant, going from −139 to −324 MHz. For comparison, the latter value is similar to that calculated for the square-planar complex [Cu(NH_3_)_4_]^2+^ (−362 MHz) using referenced configuration interaction calculations (54).

Any change in value of the Fermi contact upon a chemical perturbation to the copper coordination sphere arises from two principal sources: differences in the 4s mixing with the SOMO (a positive contributor to the value of the Fermi contact) and/or changes to spin polarization of the copper core (a negative contributor) or valence (a positive contributor) orbitals. In this case, it is unlikely the change in the value of the Fermi contact upon substrate binding is due only to a reduction in 4s orbital contribution to the SOMO, since the observed shift of +185 MHz in Fermi contact upon substrate binding would require a 4s orbital content of ∼4% in the absence of substrate (55, 56). This value is higher than expected when compared to the d(z^2^) content of ∼2%. Thus, the changes in the value of the Fermi contact upon substrate binding are also caused by a significant increase in core orbital polarization and/or reduction in polarization of valence orbitals. Previous studies on copper(II) complex hyperfine values have emphasized the importance of valence shell polarization, especially when there is a change in coordination number at the copper (with polarization of the core orbitals being relatively insensitive to coordination changes and proportional to the overall spin density at the Cu) (57). As such, the large change in Fermi contact upon substrate binding likely arises from the change in coordination number of five to four at the copper with an attendant reduction in the polarization of the valence electrons, together with a small contribution due to the reduction of 4s orbital mixing with the SOMO.

#### Substrate-induced changes in metal–ligand covalency.

**Fig. 4.**
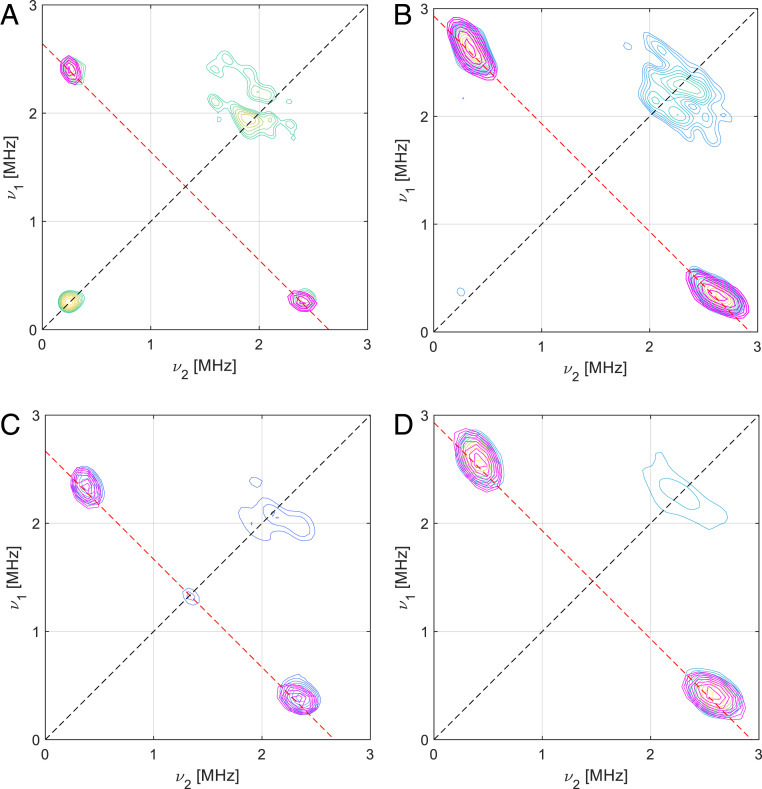
^15^N-HYSCORE spectra and simulations of ^63^Cu-^15^N-*Bl*LPMO10A (*A* and *B*) and ^63^Cu-^15^N-*Bl*LPMO10A with squid pen β-chitin (*C* and *D*). Numerical simulations (in pink) were obtained with the values reported in Table 3. (*A*) ^63^Cu-^15^N-*Bl*LPMO10A near *g*_⊥_ with τ = 136 ns at 3,395 G; (*B*) ^63^Cu-^15^N-*Bl*LPMO10A near *g*_∥_ with τ = 136 ns at 3,060 G; (*C*) ^63^Cu-^15^N-*Bl*LPMO10A with squid pen β-chitin near *g*_⊥_ with *τ* = 200 ns at 3,390 G; (*D*) ^63^Cu-^15^N-*Bl*LPMO10A near *g*_∥_ with τ = 200 ns at 3,090 G.

Further indications about any changes in metal–ligand bonding upon substrate binding can be gleaned from the values of the largest nitrogen superhyperfine coupling values. Table 1 shows that upon substrate addition, the coupling values assigned to the two Cu–^15^N(His) interactions reduce from ∼60 (±5) to ∼55 (±2) MHz, and that there is an increase in the coupling assigned to the Cu–^15^NH_2_ interaction from ∼40 (±5) to ∼45 (±2) MHz. Analogous shifts, corrected for the difference in gyromagnetic ratio, are seen for the ^14^N isotopologue. Despite the excellence of the simulated fits, however, the estimated errors in the values preclude a definitive conclusion about differences in their values before and after substrate addition. The major source of error lies in the CW-EPR spectrum of *Bl*LPMO10A in the absence of substrate. Thus, Davies ^14^N electron nuclear double resonance (ENDOR) data were collected on the enzyme before substrate addition, which were orientation-selected to the Cu perpendicular direction (*SI Appendix*, Fig. S5) (45). (Unfortunately, we were unable to collect satisfactory spectra on the sample after substrate addition; see below.) The spin-Hamiltonian parameters obtained from the ^14^N ENDOR experiments (*SI Appendix*, Table S2) match well the values derived from CW-EPR experiments, giving *A*_∥,N_(Cu,⊥) = 23 (±1) MHz [cf. 28 (±5) MHz from CW-EPR], assigned to the N terminus nitrogen, and *A*_∥,N_(Cu,⊥) = 40 (±2) MHz [cf. 43(±5) MHz from CW-EPR] for the two His nitrogen atoms, lending confidence in the values obtained from CW-EPR. (It is notable that the experimental Cu–^14^NH_2_ coupling value is much less than that obtained from previous DFT calculations on LPMOs, ∼50 MHz, likely reflecting the well-known issues with DFT in calculating accurately such coupling values.) Given the lack of a satisfactory ^14^N ENDOR spectrum of the enzyme in the presence of substrate, orientation-selective ^15^N-hyperfine sublevel correlation (HYSCORE) spectra before and after chitin addition were also collected (45). The use of ^15^N in this regard significantly simplifies the spectra, allowing extraction of the key coupling values with high accuracy (see next section for further discussion of these data). These data show that, upon substrate addition, the dipolar coupling of the two remote nitrogen atoms of each of the two imidazole rings of the histidine brace increases from T = 0.30 and 0.34 MHz to 0.35 and 0.40 MHz, respectively (Table 3 and Fig. 4), while the *a*_iso_ couplings decrease from 2.00 to 1.90 and 2.40 to 2.20 MHz. The calculated A+2T values, which represent the axial coupling of the N atoms to the Cu, can thus be estimated to be 2.6 and 3.1 MHz in the absence of substrate and 2.6 and 3.0 MHz in the presence of substrate. The small decrease in *a*_iso_ upon substrate binding (∼10%; Table 3) is in accord with the decrease in the coupling constants of the coordinating nitrogen atoms of the histidine rings observed from simulations of the CW-EPR data (Table 1). The small increase in dipolar coupling is in accord with an increase in spin density at the Cu upon substrate binding.

**Table 3.** ^15^N-HYSCORE simulation parameters for ^63^Cu(II)-^15^N-*Bl*LPMO10A with and without squid pen β-chitin

	^63^Cu*-*^15^N*-Bl*LPMO10A			^63^Cu*-*^15^N*-Bl*LPMO10A + β-chitin		
	*a*_iso_	T	A Frame Euler angles	*a*_iso_	T	A Frame Euler angles
N(A) ⊥	2.40 (0.02)	0.34 (0.03)	[10 100 60]	2.20 (0.05)	0.40 (0.02)	[10 110 60]
N(B) ⊥	2.00 (0.05)	0.30 (0.05)	[0 94 20]	1.90 (0.05)	0.35 (0.05)	[120 70 0]
N(A) ∥	2.40 (0.02)	0.34 (0.05)	[10 100 60]	2.25 (0.05)	0.40 (0.05)	[10 110 60]
N(B) ∥	2.00 (0.05)	0.30 (0.05)	[0 94 20]	1.85 (0.05)	0.35 (0.05)	[120 60 0]

The perpendicular and parallel symbols are used to define the set of simulation parameters used for the spectra collected at that field position (∥ for 3,060 or 3,090 G and ⊥ for 3,995 G). The numbers in brackets represent the error on the measurement estimated from the quality of simulated fits. The Euler angles define the *zy′z″* rotations with respect to the *g* matrix directions.

The overall picture that emerges is that substrate binding drives a structural rearrangement at the copper ion characterized by a greater Cu–NH_2_ covalency and reduced Cu–His covalency, accompanied by a small but significant increase in spin density at the Cu. Such changes would be consistent with a structural rearrangement where the N_3_ T-shaped coordination geometry provided by the histidine brace is shortened in the Cu–NH_2_ direction and elongated in the Cu–N_His_ directions upon substrate binding.

#### Substrate-induced changes to hydrogen bonding to the histidine brace.

It is known from crystal structure and modeling studies of LPMOs with oligosaccharides that the substrate does not form a direct coordination bond to the copper (22, 31, 32). Thus, the substrate-induced changes in the copper coordination sphere described above are due to changes in the primary coordination sphere brought about by the substrate or through interactions of the substrate with the copper’s outer coordination sphere(s). In terms of these outer coordination sphere interactions, N-HYSCORE spectroscopy is a powerful tool in that it can provide hydrogen-bonding information on the remote nitrogen atoms on the imidazole rings of the histidines. In particular, the values of the quadrupole tensor that are determined from ^14^N-HYSCORE spectra include an estimate of the electric field gradient that exists at the nitrogen atom, which—in turn—is directly related to the strength of any hydrogen bond formed at the N–H group.

Determining accurate values for the electric field gradient at the ring ^14^N-nitrogen atoms requires the accurate evaluation of both the quadrupole and hyperfine tensor values. This is normally difficult; however, as described above, simulation of the ^15^N HYSCORE spectra in the absence of substrate gave *a*_iso_ values of 2.40 ± 0.02 and 2.00 ± 0.05 MHz and dipolar coupling values (T) of 0.34 ± 0.03 and 0.30 ± 0.05 MHz. Upon substrate addition, the *a*_iso_ values reduce by 5 to 10% and the T values increase by ∼15% (Table 3 and Fig. 4). These accurate values for coupling could then be used in the ^14^N HYSCORE simulations (*SI Appendix*, Fig. S6), from which it was then possible to determine the quadrupole tensor elements (*e*^*2*^*qQ/h* = Κ and η), where the asymmetry parameter η is related to the strength of the hydrogen bond interaction (58). In particular, a value of η close to 1 is associated with the N*–*H participating in a strong H bond with an outside H-bond acceptor, while values between 0.45 and 0.75 are associated with weak H bonds. The nuclear quadrupole parameters for ^63^Cu-*Bl*LPMO10A before and after addition of β-chitin (Table 4) show that the H-bonding environment of one of the nitrogen atoms is not perturbed by the binding of chitin. Conversely, the η value of the other nitrogen atom increases from 0.7 to 0.9, showing that the substrate drives a significant change in the H-bond network around this N–H group, possibly through a direct H-bonding interaction with the substrate. On the basis of the DFT calculations (*SI Appendix*, Table S8), it is possible to tentatively assign the former [N(B) in Tables 3 and 4] as the remote nitrogen on His32 and the latter [N(A) in Tables 3 and 4] as the remote nitrogen on His121, in accordance with modeling studies carried out on another chitin-active LPMO (32). This change in hydrogen-bonding pattern around the histidine is likely an important contributor to the observed changes in Cu–N(His) covalency brought about by substrate addition.

**Table 4.** ^14^N-HYSCORE simulation parameters for ^63^Cu(II)-*Bl*LPMO10A with and without squid pen β-chitin

	*a*_iso_	T	A Frame Euler angles	Κ	η	Q Frame Euler angles
^63^Cu*-Bl*LPMO10A						
N(A)	1.6	0.3	[10 100 60]	1.75 (0.05)	0.7 (0.05)	[60 10 95]
N(B)	1.3	0.25	[0 94 20]	1.40 (0.05)	0.85 (0.05)	[20 0–95]
^63^Cu*-Bl*LPMO10A + β-chitin						
N(A)	1.55	0.35	[10 100 60]	1.35 (0.05)	0.9 (0.05)	[60 30 95]
N(B)	1.25	0.30	[120 70 0]	1.40 (0.05)	0.8 (0.05)	[10–10 -80]

The spectra were collected near *g*_⊥_ (3385 G and 3390 G for the sample without and with β-chitin, respectively, with τ = 200 or 136 ns). The numbers in brackets represent the error on the measurement estimated from the quality of simulated fits. The Euler angles define the *zy′z″* rotations with respect to the *g* matrix. Spectra are shown in *SI Appendix*, Fig. S6.

#### Substrate-induced changes in d-orbital energies.

To probe further the changes at the copper brought about by substrate binding, a calculation was made to determine changes in the value of *g*_iso_. Unlike *A*_iso_, this value reports on the nature of both the ground-state SOMO and its associated excited-state SOMOs (generated by Cu based d-d excitations), including the energy separations of the d orbitals and the notional metal–ligand π-covalency of the excited states. Accordingly, an analysis of *g*_iso_ is complicated and care must be exercised in its interpretation. Notwithstanding this caveat, however, the *g*_iso_ value is seen to reduce (Δ ∼ 0.03) upon substrate addition (Table 1). This reduction is counter to that expected from a decrease in either the covalency and/or d(z^2^) mixing of the ground-state SOMO, but it is commensurate with an increase in the energy separation of the SOMO from excited d orbital states (which is also reflected in a decrease in the value of *g*_3_ by ∼0.05 upon substrate addition). Without access to electronic absorption data, which is precluded by the solid nature of the chitin substrate, it is not possible to be more definitive about the d-d transition energies. However, in an indication of the adoption of a more axial-like symmetry upon substrate binding, the difference in values of *g*_1_ and *g*_2_, Δ*g*, reduces from ∼0.06 to ∼0.01. A large Δ*g* is associated with d(z^2^) mixing, which would arise from the distorted coordination geometry before substrate addition. The greater degree of d(z^2^) character in the SOMO before substrate addition is reproduced by the DFT calculations (2.7% compared to 0.5% following substrate addition; see below and *SI Appendix*, Table S9). Therefore, the changes in *g* values are ones that would be expected when the copper coordination sphere rearranges from a distorted square pyramid (five-coordinate) to one which is near axial square planar (four-coordinate), a geometry change that would be accompanied by an increase in the relative energy (with respect to the other d orbitals) and orbital character of the d(x^2^−y^2^) SOMO.

Overall, the electronic changes to the copper SOMO in chitin-active AA10 LPMOs that occur upon addition of substrate are as follows: a reduction in the asymmetry of the equatorial plane of the copper coordination sphere [likely associated with a smaller difference in the Cu–N(His) to Cu–N(amino) distances upon chitin addition], an increase in the relative energy of the SOMO, a reduction in ground-state metal–ligand covalency to the histidine ligands, and an increase in the d(x^2^−y^2^) character of the SOMO through a process of reduced interactions with metal and ligand-based orbitals. A simpler view of these changes would be that, before substrate addition, the redox-active orbital on the copper is somewhat delocalized through mixing with other orbitals on both the metal and ligands. However, the addition of substrate allows for a spatially contracted and energetically well-defined d(x^2^−y^2^) orbital to “surface” at the copper, one that is capable of forming a strong covalent interaction with an exogenous ligand in the equatorial plane of the copper coordination sphere. The mechanistic consequences are discussed further below, but such a switch in the character and energy of the frontier redox-active orbital clearly provides a basis for a potential coupling mechanism between the substrate and any exogenous ligands on the copper (e.g., O_2_^−^).

### DFT Calculations.

**Fig. 5.**
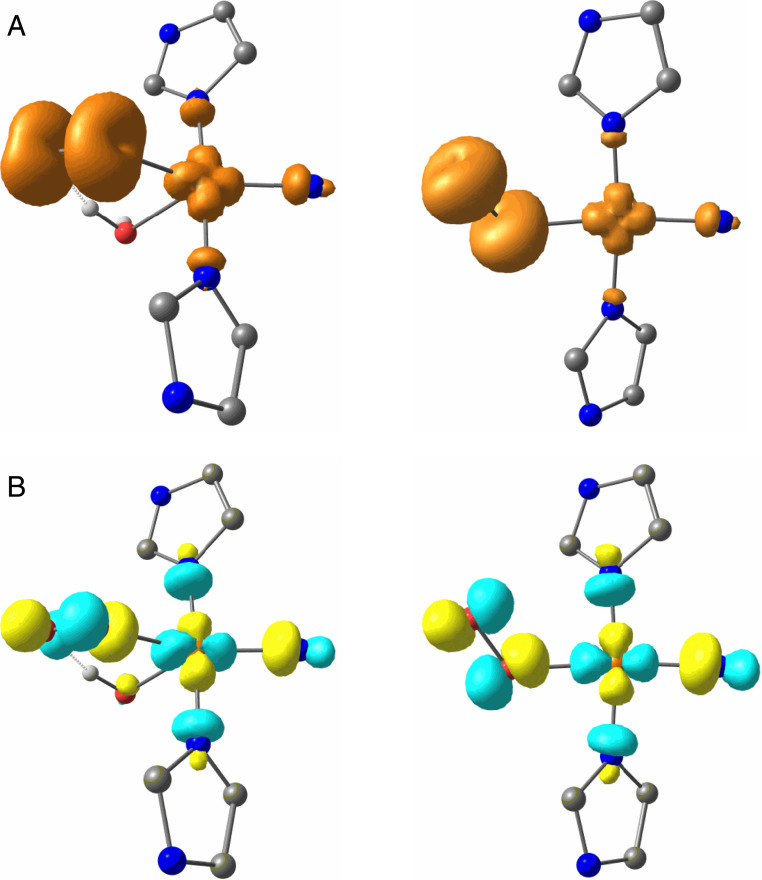
(*A*) Spin-density contour and (*B*) α-HOMO electron density (with wavefunction phase depicted in color) contour plots of the five-coordinate (*Left*) and four-coordinate (*Right*) copper–superoxide complexes within the active site of AA10 LPMOs.

To corroborate this analysis, DFT calculations were performed on the closely related *Ba*AA10 LPMO, as described above, for two different models of the active site (see *SI Appendix*, *Supplementary Discussion*, for details). The first, which emulates the enzyme in the absence of substrate, contained a superoxide and a water molecule in the copper’s coordination sphere in addition to the coordinating atoms of the histidine brace, and the second, emulating the enzyme in the presence of substrate, contained only superoxide as the exogenous ligand. From each calculation, spin population analysis (Fig. 5 and *SI Appendix*, Table S9) reveals that the switch from five-coordinate to four-coordinate Cu(II) is accompanied by a large decrease in spin population on Cu(II) from 54 to 41%, which transfers almost completely to the distal oxygen atom of the superoxide [without a significant change in the O–O bond length, Δr(O–O) = 0.02 Å]. These changes in spin population therefore reflect the high degree of covalency between the copper and the superoxide ligand in the four-coordinate state, i.e., when substrate is bound (Fig. 5), corroborating the foregoing DELFT analysis of the changes that occur at the active site on the addition of substrate to *Bl*LPMO10A.

It was further possible from these calculations to estimate the relative change in the strength of the Cu(II)–superoxide bonds upon substrate addition (*SI Appendix*, Table S10 and Figs. S10 and S11). This estimation is made by performing single-point optimizations of the active sites in the presence and absence of superoxide (*SI Appendix*, Fig. S11), and then calculating the difference in electronic energies between the two for both five-coordinate Cu (Δ*E*_1_) and four-coordinate Cu (Δ*E*_2_) cases. ΔΔ*E* (=Δ*E*_1_ − *E*_2_) can then be calculated in which all intrinsic errors in the calculated point electronic energies, save for a small basis set superposition error, are expected to cancel leaving only the difference in copper–superoxide bond strength as the principal contributor to the value of ΔΔ*E*. This value shows that the Cu(II)–superoxide bond is ∼8.2 kcal⋅mol^−1^ greater in the four-coordinate substrate-bound case. Translated into equilibrium constant terms at 298 K, where it is assumed that ΔΔ*G* ∼ ΔΔ*E*, this difference means that—in the presence of substrate—the copper–superoxide complex is ∼10^6^ more stable to dissociative elimination than in the absence of substrate.

It is expected that the thermodynamic stabilization of the Cu(II)–superoxide intermediate is enhanced by the lack of a water molecule in the copper coordination sphere, which is only the case when the substrate is bound. Such a complex can be expected to have a longer lifetime that the equivalent one in the absence of substrate where both a water molecule and superoxide coordinate to the copper ion. In this case, as shown previously by Kjaergaard et al. (14) for AA9 LPMOs from stopped flow experiments and DFT calculations, the superoxide can be expelled from the copper coordination sphere with an activation barrier of ∼10 kcal⋅mol^−1^, although this value is quite a lot lower than that recently calculated by Caldararu et al. (59) in QM/MM calculations for superoxide release from an AA10 LPMO at 19 kcal⋅mol^−1^. Our own relaxed–surface-scan DFT calculations for *Bl*LPMO10A indicate, in the absence of substrate, that a water-assisted superoxide dissociation from Cu(II) is indeed feasible with an activation barrier of only ∼4 kcal⋅mol^−1^ (*SI Appendix*, Fig. S10), similar to the experimental findings of Kjaergaard et al. Thus, the Cu(II)–superoxide complex in the absence of substrate appears to be unstable. A coupling mechanism between substrate binding and selective O_2_ activation in AA10 LPMOs therefore emerges from this analysis, in which the Cu(II)–superoxide intermediate is kinetically unstable to dissociative elimination in the absence of substrate [possibly followed by reduction of the Cu(II) and formation of O_2_] but is thermodynamically stabilized in the presence of substrate.

### Mechanism of Substrate and O_2_ Coupling in Chitin-Active AA10 LPMOs.

**Fig. 6.**
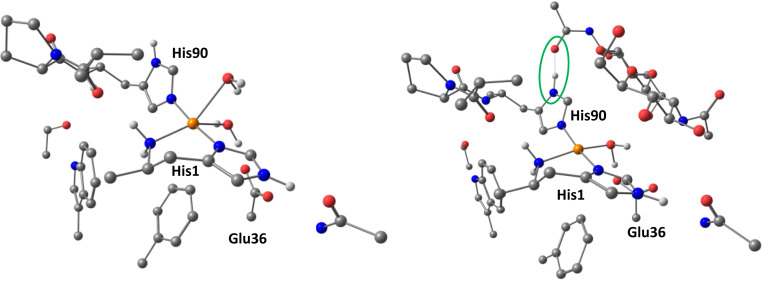
DFT-optimized structures of absence and presence of substrate in the active site of *Ba*AA10, highlighting the change in Cu coordination geometry in going from five to four ligands. All hydrogen atoms apart from those on the N and O atoms of the metal ligands were hidden for clarity.

The combined EPR data show that addition of chitin results in significant changes in the copper d-orbital electronics. This change in electronics can be explained by the formation of a more axial coordination geometry, and—in what is the most significant perturbation to the electronics of the copper brought about by chitin binding—a large increase in the relative energy/d(x^2^−y^2^) character of the SOMO, accompanied by a small reduction in metal–histidine covalency, the latter driven by formation of a hydrogen bond between substrate and a histidine and a reduction in the coordination number of the copper from five to four (Fig. 6). Importantly, within the context of O_2_ activation at the copper, any reduction in the covalency of the RAMO (redox-active MO) [which is the doubly occupied d(x^2^−y^2^) in the Cu(I) oxidation state] coupled to an increase in its relative energy, increases the relative stability of a Cu(II)-superoxide by 1) reducing the energy gap between the SOMO of Cu(II) and the π* antibonding orbital of O_2_^−^ (such a strong bond is needed to offset the negative reduction potential of O_2_ to O_2_^−^), and 2) maximizing the stabilizing effects of the nephelauxetic expansion that occurs upon the formation of a covalent bond between O_2_^−^ and Cu(II). This latter effect reveals the contributing role of electron–electron repulsions at the copper within a histidine brace coordination, which—upon formation of a Cu(II)–superoxide—results in a net transfer of spin density from the Cu(II) to the distal atom of the superoxide (*SI Appendix*, Table S9), while maintaining the superoxide character of the ligand, Δ*r*(O–O) = −0.02 Å. Such an increase in the spin density at this oxygen atom would be in accord with it acting as the site of hydrogen atom transfer from the substrate and would further contribute to any coupling mechanism induced by the substrate.

## Conclusions

The NMR structures of the *apo*- and Cu(I)- forms of *Bl*LPMO10A were determined in order to provide structural information on LPMOs in solution. There are minimal differences between the Cu(I)- and *apo*- structures of *Bl*LPMO10A. These differences are centered around the LPMO copper site and are likely related to the structural effects of copper binding. In addition, the PRE effect in NMR spectra of Cu(II)-*Bl*LPMO10A was evaluated using parameters calculated from EPR data (*g*_iso_) and derived from heteronuclear relaxation data, and shown to be consistent with PREs calculated from *Bl*LPMO10A structures. Multifrequency CW-EPR spectroscopy enabled the determination of accurate spin-Hamiltonian values, showing that the addition of chitin drives a rearrangement of the copper coordination sphere from five- to four-coordinate, accompanied by a reduction in metal–histidine covalency and the associated generation of a high-energy SOMO with significant d(x^2^−y^2^) character. This orbital essentially emerges as a well-defined frontier orbital at the copper, which can then form strong interactions with exogenous ligands such as O_2_^−^. These changes at the copper upon substrate binding provide a means by which the formation of a Cu(II)–superoxide can be stabilized. Overall, these results show that the mechanism of substrate–O_2_ coupling can be effected through rearrangement of the copper coordination geometry and subsequent changes in the d-orbital electronics, with minimal change in the rest of the protein backbone structure. These results underline recent observations in other copper proteins (60) that minimal structural changes can be coupled to large electronic changes at the copper active site.

In a wider observation on the mechanisms of LPMOs, this work shows that substrate binding is coupled to the activation of the O_2_ cosubstrate. For LPMOs, with their exposed copper sites, substrate-induced activation of the catalytic center is an attractive scenario since this will reduce off-pathway reactions that may lead to enzyme inactivation. As such, any studies on LPMOs must take into account the fact that specific LPMOs may be associated with specific substrates, an association through which the “on-pathway” coupled mechanism operates. As a caution, therefore, investigators need to be aware that any studies performed on LPMOs not correctly bound to their natural substrate may not have an on-pathway mechanism available to them, potentially leading to rapid enzyme inactivation via indiscriminate redox chemistry. Finally, the results presented in this current study demonstrate the power of an integrated NMR/EPR spectroscopic approach to studying LPMOs.

## Materials and Methods

Detailed information for all experimental procedures is provided in *SI Appendix, SI Materials and Methods*.

### Sample Preparation.

Isotope-labeled and nonlabeled *Bl*LPMO10A were recombinantly produced in *E. coli* using isotope-enriched (^13^C, ^15^N) minimal medium or LB, respectively, and purified by multiple chromatographic steps as described previously (38, 39, 61).

### Enzyme Activity.

Substrate degradation was performed using standard reaction conditions and product formation was analyzed using hydrophilic interaction chromatography, as described by Loose et al. (62). Reactions were set up with 10 mg/mL α- or β-chitin, 1 μM of Cu(II)-loaded *Bl*LPMO10A, and 2 mM ascorbic acid in 20 mM Tris⋅HCl buffer (pH 8.0) in a shaking incubator at 40 °C.

### NMR Spectroscopy.

NMR spectra of *Bl*LPMO10A were recorded on Bruker Avance III 600- and 800-MHz spectrometers. Three-dimensional NOESY-edited spectra were recorded, assigned, and integrated. NOE cross-peak intensities were converted to distance restraints and were used together with dihedral torsion angles predicted by TALOS-N (63) as input for structure calculations in CYANA (64). The 20 conformers with lowest CYANA target function values were energy-minimized using YASARA (65). The *apo*-*Bl*LPMO10A ensemble was deposited in PDB under ID code 5LW4. The Cu(I)-*Bl*LPMO10A ensemble was deposited in PDB under ID code 6TWE. Cu(II) PREs were measured by analyzing ^15^N-HSQC signal intensities before and after addition of Cu(II) to *apo*-*Bl*LPMO10A. PREs were calculated from the structural ensemble.

### EPR Spectroscopy.

EPR experiments were recorded on *Bl*LPMO10A and ^15^N-*Bl*LPMO10A loaded with ^63^Cu(II), with and without squid pen β-chitin. CW X-band spectra were acquired at 165 K on a Bruker EMX spectrometer operating at ∼9.30 GHz, with modulation amplitude of 4 G, modulation frequency of 100 kHz and microwave power of 10.02 mW. CW Q-band spectra were acquired at 113 K on a Jeol JES-X320 spectrometer operating at ∼34.7 GHz, with modulation width 0.8 mT and microwave power of 1.0 mW. HYSCORE spectra were collected on a Bruker ElexSys E580 spectrometer equipped with an Oxford CF 935 helium flow cryostat. The rf pulse in Davies ENDOR spectra was generated by the Bruker DICE system and amplified by a 60-dB gain ENI A-500-W amplifier. Spectral simulations were carried out using EasySpin 5.2.6 (66) integrated into MATLAB R2017a software.

## Supplementary Material

Supplementary File

## Data Availability

The data for NMR structures and their restraints have been deposited in the Research Collaboratory for Structural Bioinformatics Protein Data Bank (https://www.rcsb.org/) under the following PDB ID codes: 5LW4 (*apo*-BlLPMO10A) and 6TWE (Cu^+1^-*Bl*LPMO10A). Data and code for PRE calculations (Cu^+2^-*Bl*LPMO10A) are available at https://github.com/gcourtade/BlLPMO10A. Raw CW-EPR data can be found at https://pure.york.ac.uk/portal/en/datasets/spectroscopic-investigation-of-blaa10-lpmo(969dd5ce-c1fa-47f4-ba56-e0b026050ed0).html. All other data are available in the main text and *SI Appendix*.
