# Supplementary material for: Mechanistic basis of substrate–O2 coupling within a chitin-active lytic polysaccharide monooxygenase: An integrated NMR/EPR study
Source: Proc Natl Acad Sci U S A. 2020 Jul 28;117(32):19178–89. doi: 10.1073/pnas.2004277117 (PMC7431007; doi:10.1073/pnas.2004277117)
Supplement: Supplementary File [file pnas.2004277117.sapp.pdf]

SI Appendix for

Mechanistic basis of substrate-O_2 coupling within a chitin-active lytic polysaccharide monooxygenase: an integrated NMR/EPR study

Gaston Courtade[a,1], Luisa Ciano[b,c,d,1,2], Alessandro Paradisi[b], Peter J. Lindley[b], Zarah Forsberg[e], Morten Sørlie[e]; Reinhard Wimmer[f], Gideon J. Davies[b], Vincent G. H. Eijsink[e], Paul H. Walton[b,3], Finn L. Aachmann[a,3]

[1] G.C. and L.C. contributed equally to this work.

Author affiliation

[a] NOBIPOL, Department of Biotechnology and Food Science, NTNU Norwegian University of Science and Technology, Sem Sælands vei 6/8, N-7491 Trondheim, Norway

[b] Department of Chemistry, University of York, Heslington, York YO10 5DD, UK

[c] School of Chemistry and Photon Science Institute, University of Manchester, Oxford Road, Manchester, M13 9PL, UK

[d] Photon Science Institute, University of Manchester, Manchester M13 9PL, United Kingdom;

[e] Faculty of Chemistry, Biotechnology and Food Science, NMBU Norwegian University of Life Sciences, N-1432 Ås, Norway

[f] Department of Chemistry and Bioscience, Aalborg University, Frederik Bajers vej 7H, 9220 Aalborg Ø, Denmark

[2]Current address: School of Chemistry, University of Nottingham, University Park, Nottingham, NG7 2RD, UK

Corresponding authors

[3] To whom correspondence should be addressed.

Finn L. Aachmann

NOBIPOL, Department of Biotechnology and Food Science, NTNU Norwegian University of Science and Technology, Sem Sælands vei 6/8, N-7491 Trondheim, Norway

Tel.: +47 73593317

Paul H. Walton

Department of Chemistry, University of York, Heslington, York YO10 5DD, UK

Tel: +44 1904 324457

E-mail: finn.l.aachmann@ntnu.no, paul.walton@york.ac.uk

www.pnas.org/cgi/doi/10.1073/pnas.2004277117

TABLE OF CONTENTS

SI Materials and Methods

Supplementary Discussion – Calculations used in EPR analysis

Supplementary Discussion - DFT calculations in presence of substrate

Table S1 – Structural statistics of *apo-Bl*LPMO10A

Table S2 – Davies ^{14}N ENDOR simulation parameters for *Bl*LPMO10A

Table S3 – The functional and basis set schemes used to calculate the EPR properties of *Bl*LPMO10A

Table S4 – Calculated spin Hamiltonian parameters for the 'resting state' Model A of *Bl*LPMO10A

Table S5 – Calculated spin Hamiltonian parameters for '4-coordinate' Model B of *Bl*LPMO10A

Table S6 – Calculated spin Hamiltonian parameters for 'substrate bound' Model C of *Bl*LPMO10A

Table S7 – EPR properties of *Bl*LPMO10A with and without β-chitin

Table S8 – EPR properties of the remote nitrogen atoms of the histidine rings of *Bl*LPMO10A with and without β-chitin, determined experimentally and by DFT calculations

Table S9 – Löwdin spin population analysis of 4-coordinate and 5-coordinate superoxide models (adding up to 200% for a total of 2 spins).

Table S10 – Calculated superoxide binding energies using cluster models (D) and (E).

Figure S1 – *Bl*LPMO10A activity and binding towards chitin

Figure S2 – Effect of Cu(II)-binding on *apo-Bl*LPMO10A

Figure S3 – Backbone dynamics of *apo-Bl*LPMO10A

Figure S4 – X and Q band CW-EPR spectra

Figure S5 – Davies ^{14}N ENDOR spectra of ^{63}Cu-*Bl*LPMO10A

Figure S6 – ^{14}N HYSCORE of ^{63}Cu-*Bl*LPMO10A

Figure S7 – Starting structure for geometry optimization of Cu(II)-*Bl*LPMO10A

Figure S8 – Geometry optimized structure of Cu(II)-*Bl*LPMO10A

Figure S9 – Geometry optimized structure of Cu(II)-*Bl*LPMO10A in the 'substrate-bound' case

Figure S10 – Superoxide release from Cu(II)-AA10. DFT-optimized structures with Cu-O distances fixed at 2.20, 2.50 and 3.00 Å.

Figure S11 – Geometry optimized structures of 4-coordinate and 5-coordinate Cu(II)-*Bl*LPMO10A-superoxide cluster models and resulting geometries following superoxide release.

References

Appendix – Atom coordinates for DFT models

Materials and Methods

Protein production and functional characterization

Production and purification of *Bl*LPMO10A were performed as previously described (1). Copper saturation of purified protein was achieved by incubation with a 3-fold molar surplus of Cu(II)SO_4 at room temperature for 30 min followed by removal of excess copper using a PD MidiTrap G-25 desalting column (GE Healthcare; Uppsala, Sweden) equilibrated with 20 mM Tris/HCl buffer pH 8.0 (2). Analysis of oxidized chito-oligosaccharides released in reactions with ball-milled shrimp shell (*Pandalus borealis*) α-chitin with a particle size of ~µm (Chitinor AS; Senjahopen, Norway) or ball milled squid β-chitin with a particle size of < 0.85 mm (France chitin; Orange, France) was performed using hydrophilic interaction chromatography (HILIC) as described by Loose *et al.* (2). The enzymatic reaction mixtures contained 1 µM of Cu(II)-loaded *Bl*LPMO10A, 10 mg/mL suspension of α- or β-chitin, 20 mM Tris/HCl buffer, pH 8.0, and 2 mM ascorbic acid. Reactions were incubated at 40 °C in an Eppendorf Thermomixer set to 800 rpm for 24 hours.

Binding experiments were performed using 2 µM of Cu(II)-loaded *Bl*LPMO10A and 10 g/L β-chitin. The binding reactions was carried out in 50 mM sodium phosphate buffer pH 7.0 and incubated at 40 °C in an Eppendorf Thermomixer set to 800 rpm. At various time points (0, 5, 15, 30, 60, 120 and 240 min) a sample was taken and filtrated, using a 96-well filter plate (Millipore) operated by a Millipore vacuum manifold, to remove insoluble substrate and substrate-bound protein. The concentration of protein in the supernatant (e.g. non-substrate bound protein) was determined using the Bradford assay and measuring A_{595} in an Eppendorf Biophotometer (Eppendorf, Hamburg).

Sample preparation for NMR

Cloning, protein production using an LPMO expression cassette, and purification of the natural abundance and isotope-labeled (^{15}N and ^{13}C or ^{15}N) *Bl*LPMO10A from *Bacillus licheniformis* (UniProt entry Q62YN7, residues 32-203), as well as conditions for NMR spectroscopy measurements, have been described previously (3, 4). In order to obtain the *apo* form of *Bl*LPMO10A, protein samples were incubated in 25 mM sodium phosphate, pH 5.5, containing 10 mM NaCl and 8 mM EDTA at 4 °C overnight, prior to exchanging the buffer to either 25 mM sodium phosphate, pH 5.5, 10 mM NaCl, or 20 mM MES, pH 5.5, using Vivaspin 6 spin-columns (5 kDa cut-off, Sartorius).

NMR samples with Cu(II) were prepared and recorded in the following way. First, the buffer of a solution of ^{13}C- and ^{15}N-labeled *apo-Bl*LPMO10A was changed to a 25 mM sodium acetate buffer, pH 5.5, 10 mM NaCl. The protein solution was then concentrated to 0.1 mM and ~450 µL, and reference spectra (1D-proton, ^{15}N-HSQC and aromatic ^{13}C-HSQC) were recorded. Then, $CuSO_4$ was added to concentrations of 0.15 mM, and spectra were recorded with $CuSO_4$ present.

NMR samples with Cu(I) were prepared and recorded in the following way. First, the buffer of a solution of ^{13}C- and ^{15}N-labeled *apo-Bl*LPMO10A was changed to 20 mM MES, pH 5.5, followed by concentrating the protein solution to 0.1 mM and ~450 µL, after which reference spectra (1D-proton and ^{15}N-HSQC) were recorded. Then, the sample was incubated for 48 h in BBL GasPak Jar (Becton Dickinson, NJ, USA) chamber with a BBL CO_2 Generator bag (Becton Dickinson, NJ, USA) to remove oxygen gas. The chamber was transferred to a Whitley A45 Anaerobic Workstation (Don Whitley Scientific Limited, UK), where the protein sample was transferred to a 5 mm NMR tube and Cu(I) was added in the form of a pellet (appr. 1 mg) of Cu(I)Cl. Finally, the tube was sealed with a rubber septum and parafilm. NMR spectra (1D-proton, ^{15}N-HSQC, aliphatic ^{13}C-HSQC, aromatic ^{13}C-HSQC, ^{15}N-edited-NOESY-HSQC and ^{13}C-edited-NOESY-HSQC) were recorded immediately after.

NMR spectroscopy

NMR spectra of *Bl*LPMO10A were recorded at 25 °C on a Bruker Avance III 600 MHz or Avance III 800 MHz spectrometer, both equipped with a 5 mm Z-gradient CP-TCI (H/C/N) cryoprobe, at the NT-NMR-Center/ Norwegian NMR Platform in Trondheim, Norway, and on a Bruker Avance III 600 MHz equipped with a 5 mm Z-gradient Prodigy TCI (H/C/N) cryoprobe, at the Department of Chemistry and Biosciences, Aalborg University, Aalborg, Denmark. NMR data were processed using Bruker TopSpin version 3.5. NMR spectral analysis was performed using CARA version 1.5.5 (5). The NMR assignment of *Bl*LPMO10A has been published elsewhere (3). For structure determination, three-dimensional ^{13}C-edited and ^{15}N-edited NOESY-HSQC spectra, as well as ^{1}H-^{1}H NOESY spectra were recorded. NOE cross-peaks were manually identified, assigned, and integrated using the NEASY program within CARA version 1.5.5 (5). The {^{1}H}-^{15}N heteronuclear NOEs were derived with Protein Dynamic Center software version 2.3.3 from Bruker BioSpin using two independently measured and integrated ^{15}N-heteronuclear correlated spectra with and without ^{1}H saturation (6). Nuclear magnetic relaxation time measurements of ^{15}N nuclei (T_1 and T_2) were analyzed with Protein Dynamic Center software version 2.3.3 from Bruker BioSpin, where the data from ^{15}N-HSQC-type spectra acquired with different relaxation delays was exponentially fitted (6, 7). The rotational

correlation time was estimated using the T_1/T_2 ratio, assuming overall isotropic tumbling of the protein. Secondary structure elements were analyzed using the web-based version of the TALOS-N software spin.niddk.nih.gov/bax/nmrserver/talosn/ (8) using selected chemical shifts (N, H^N,C',C^α, C^β, H^α and H^β).

PRE calculation

The PRE effect was calculated using the atomic coordinates for the 20-conformer ensemble of Cu(I)-*Bl*LPMO10A (PDB ID: 6TWE) as input. Transverse ($R_{2,para}$) PRE rates are described by the Solomon-Bloembergen equation (9, 10):

$$R_{2,para} = \frac{1}{15}\left(\frac{\mu_0}{4\pi}\right)^2 \gamma_I^2 g^2 \mu_B^2 s_e(s_e+1)[4J_{SBMF}(0) + 3J_{SBMF}(\omega_I)]$$

where μ_0 is the permeability of vacuum, γ_I is the proton gyromagnetic ratio, g is the electron g-factor, μ_B is the free electron magnetic moment, s_e is the paramagnetic electron spin number ($s_e = 1/2$ for Cu(II)), $\frac{\omega_I}{2\pi}$ is the Larmor frequency of the proton, and $J_{SBMF}(\omega)$ is the model-free (11) extension generalized spectral density function, defined by the following equation:

$$J_{SBMF}(\omega) = \langle r^{-6} \rangle \left[\frac{S^2\tau_c}{1+\omega^2\tau_c^2} + \frac{(1-S^2)\tau_t}{1+\omega^2\tau_t^2}\right]$$

where r is the distance between the proton and the paramagnetic electron, S^2 is the square of the generalized order parameter (see (12) for details regarding its calculation), τ_c is the overall protein rotational correlation time, and τ_t is the total correlation time defined as $(\tau_c^{-1} + \tau_i^{-1})^{-1}$, where τ_i is the correlation time for the internal motion.

The calculated reduction in signal intensity $\frac{I_{para}}{I_{dia}}$ can then be calculated using the following equation:

$$\frac{I_{para}}{I_{dia}} = \frac{R_{2,dia}e^{-R_{2,para}t}}{R_{2,dia} + R_{2,para}}$$

where $R_{2,dia}$ is the transverse relaxation rate in the absence of Cu(II) and *t* is the length of the ^1H transverse relaxation evolution during INEPT coherence transfers in ^{15}N-HSQC.

For *Bl*LPMO10A, *g* was set to 2.13, which is the average of the calculated g_{iso} (**Table 1**); $R_{2,dia}$ was set to 12.5 s^{-1}, which is the average of $1/T_2$ (**Figure S3**); τ_c was set to 10.2 ns, calculated from the average T_1/T_2 (**Figure S3**); τ_i was set to 500 ps. The PRE calculation was performed

using a script available at https://github.com/gcourtade/BlLPMO10A. Part of the script uses code available from https://github.com/KULL-Centre/DEERpredict.

NMR structure calculation

NOE cross-peak intensities were converted into distance restraints using the CALIBA (13) subroutine in CYANA 3.97 (13, 14). Dihedral torsion angles (φ, ψ) predicted by TALOS-N (8) were included as conformational restraints, as was one disulfide bridge (Cys45–Cys56). Based on this input, the structure was calculated using the torsion angle dynamics program CYANA 3.97 (14). The structure calculation started by generating 200 conformers with random torsion angles, and the dihedral angles in each conformer were optimized using simulated annealing in 10,000 steps, to fit the restraints. The 20 conformers with the lowest CYANA target function values were energy-minimized using YASARA (15), first *in vacuo*, followed by using water as the explicit solvent and calculating electrostatics by applying the particle mesh Ewald method (16). In both these steps the YASARA force field (17) was applied. The coordinates of the minimized *apo*-*Bl*LPMO10A conformers have been deposited in the Protein Data Bank under the ID 5LW4.

The Cu(I) structure of *Bl*LPMO10A was generated in YASARA in the following way: (i) ^{15}N-edited and ^{13}C-edited NOESY-HSQC spectra that had been recorded in the presence of Cu(I) were analyzed, integrated and converted to distance constraints. (ii) A constraint for copper was introduced by modelling a copper atom that was placed arbitrarily equidistant from the active site histidine atoms (N-His32, $N^{\delta1}$-His32, $N^{\varepsilon1}$-His121). Its geometry was constrained using partial charges for the copper-site histidines and force constraints for the bonds and angles describing the copper-histidine brace interaction, obtained by Bissaro *et al.* (18) from a minimal model of the truncated histidine brace of *Sm*LPMO10A. (iii) These new contraints were incorporated to each of the 20 conformers from the *apo*-*Bl*LPMO10A ensemble by running 100 ps of restrained molecular dynamics in YASARA to produce the Cu(I)-ensemble. The YASARA macro used for the calculation is available at https://github.com/gcourtade/BlLPMO10A, The coordinates of the Cu(I)-*Bl*LPMO10A conformers have been deposited in the Protein Data Bank under the ID 6TWE.

EPR spectroscopy

Preparation of ^{63}Cu(II) stock

A 30% solution of H_2O_2 (1 mL) was very slowly added to 300 μL of conc. H_2SO_4 cooled in ice. Pure ^{63}Cu sheets (60 mg, purchased from Sigma Aldrich) were added to this solution, which turned progressively blue. After 36 h, the solution was decanted and the undissolved copper metal was added to a freshly prepared H_2O_2/H_2SO_4 mixture. All the copper was dissolved after a further 18 h period. The two solutions were combined and bright blue crystals appeared within 48 h, which were then filtered, washed with EtOH and dried.

CW and HYSCORE EPR experiments

The *apo* forms of *Bl*LPMO10A and ^{15}N-*Bl*LPMO10A were copper loaded by addition of 0.9 equivalents of $^{63}CuSO_4 \cdot 5H_2O$ (prepared as described above) in water. Continuous wave (CW) X-band frozen solution EPR spectra of 0.29 mM and 0.17 mM solutions of ^{63}Cu(II)-*Bl*LPMO10A and ^{63}Cu(II)-^{15}N-*Bl*LPMO10A, respectively, in 10% *v/v* glycerol at pH 5.5 (20 mM MES buffer) and 165 K with and without squid pen β-chitin were acquired on a Bruker EMX spectrometer operating at ~9.30 GHz, with modulation amplitude of 4 G, modulation frequency of 100 kHz and microwave power of 10.02 mW (4 scans). (Note: absence of glycerol did not affect the EPR spectra). CW Q-band frozen solution spectra of a 1.3 mM solution of ^{63}Cu(II)-^{15}N-*Bl*LPMO10A with and without squid pen β-chitin at pH 5.5 (20 mM MES buffer) and 113 K were acquired on a Jeol JES-X320 spectrometer operating at ~34.7 GHz, with modulation width 0.8 mT and microwave power of 1.0 mW (8 scans).

Spectral simulations were carried out using EasySpin 5.2.6 (19) integrated into MATLAB R2017a software. Simulation parameters are given in **Table 1**. g_3 and $|A_3|$ values were determined accurately from the absorptions at low field. It was assumed that *g* and A tensors were axially coincident. Accurate determination of the g_1, g_2, $|A_1|$ and $|A_2|$ was obtained by simultaneous fitting of both X and Q band spectra. The spectra obtained upon addition of β-chitin presented a mixture of free enzyme and enzyme bound to the substrate. Spectra were collected up to 3 days after addition of chitin, but the free:bound ratio did not improve over time. The simulations of the chitin-bound form of the enzyme were performed after subtraction of the normalized *Bl*LPMO10A or ^{15}N-*Bl*LPMO10A (40% and 30% of free enzyme present in the samples, respectively) from the corresponding spectra.

Hyperfine sublevel correlation (HYSCORE) spectra were collected on a 1.7 mM sample of ^{63}Cu-^{15}N-*Bl*LPMO10A or a 1.8 mM sample of ^{63}Cu-*Bl*LPMO10A before and after addition of squid

pen β-chitin in 20 mM sodium phosphate buffer pH 5.5 on a Bruker ElexSys E580 spectrometer equipped with an Oxford CF 935 helium flow cryostat. The ^{15}N HYSCORE spectra were recorded near parallel (3060 or 3090 G) and near perpendicular (3395 G) directions employing the sequence $\pi/2$ - τ - $\pi/2$ - t_1 - π - t_2 - $\pi/2$ - τ – echo with τ = 136 ns for the resting state and τ = 200 ns for the substrate-bound spectra at 20 K, collecting 256 data points in both dimensions. The ^{14}N HYSCORE spectra were recorded near parallel (3090 G) and near perpendicular (3390 G) directions employing the same sequence reported above with τ = 136 ns and τ = 200 ns at 5 K or 20 K, collecting 256 data points in both dimensions. The relaxation decay was subtracted by baseline corrections (fitting by polynomials of 3-4 degrees) in both time domains, subsequently applying apodization (Hamming window) and zero-filling to 1,024 data points in both dimensions. After 2D Fourier transformation, the spectra were simulated using EasySpin (19).

The Davies ENDOR spectra were obtained using the sequence $\pi - T - \pi/2 - \tau - \pi - \tau -$ echo with mw pulses of length $t_{\pi/2}$ = 32/128 ns and t_{π} = 64/256 ns. During time T a radio frequency (rf) pulse of length t_{rf} = 12 μs was generated by the Bruker DICE system and amplified by a 60 dB gain ENI A-500 W amplifier.

Raw EPR data are available on request through the Research Data York (DOI: 10.15124/969dd5ce-c1fa-47f4-ba56-e0b026050ed0).

Computational Details

Geometry optimization

Atomic coordinates of *Ba*AA10 were obtained from the crystal structure (PDB:5IJU, resolution 1.7 Å) from the Carbohydrate-Active enZYmes (CAZy) database) (20). Included in the truncated models were the central Cu(II) ion and 9 residues (His28, Glu68, Gln92, Ala123, Pro124, His125, Thr127, Trp187 and Phe196 (numbering starting at the first histidine is position 28; His 28 and His 125 are analogous to His32 and His121 in *Bl*LPMO10A). Hydrogens were added to appropriate positions and the following modifications were made to decrease the computational cost of the calculations: His28 and His125 were truncated at the carbonyl carbon, which was replaced by a methyl group, Glu68 and Gln92 were truncated with methyl substitution of the Cγ, the nitrogen of the amide bond between Ala123 and Thr122 was replaced by methyl groups, Trp187 and Phe196 were truncated with methyl substitution of the Cβ, Thr127 was truncated

with methyl substitution of the Cα and the methyl group of Cβ was removed. For the 'resting state' model, three water molecules were retained from the crystallographic coordinates, including the two coordinating water molecules, and the nearby 'distal' water molecule (O498, O454 and O526 respectively). To mimic substrate binding, a second model was made, where the water molecules from the crystal structure were replaced by one single equatorially coordinating water molecule. In order to account for the structural constraints imposed by the protein, multiple atoms were kept frozen throughout the optimization; these atoms are denoted by asterisks in Fig. S7.

Geometry optimizations were performed using the Gaussian 09 software package. Optimizations of both models were carried out using the generalized gradient approximation (GGA) functional uBP86. Ahlrichs's Def-2-TZVP basis set was used to treat the copper, the first coordination sphere nitrogen atoms and the oxygen atoms of the copper-ligating water molecules. On all remaining atoms a Def2-SVP basis set was used. Solvation effects were accounted for using the polarizable continuum model with a dielectric constant of 80.0, as implemented by Gaussian 09. Empirical dispersion corrections were accounted for using Grimme's empirical dispersion (GD3). The resulting coordinates from the geometry optimizations of the 'resting state' (A) and the '4-coordinate' (B) models are shown by the pink wires in Fig S8 and compared to the crystallographic coordinates (blue sticks).

An additional model was constructed (based on model B) to include a short chitin oligosaccharide (NAG_2) bound to the enzyme. The substrate molecule was positioned in accord with the findings from Bisarro *et al.* (21), with particular focus on the distance of the remote nitrogen on His125 to the carbonyl of an *N*-acetyl group. Only two sugar units were included in this calculation to minimize computational expense. The substrate molecule was held in place by keeping the methyl carbon atoms of the N-acetyl groups and the terminal oxygen atoms (in the 1 and 4 positions) frozen. The resulting structure is shown in Fig. S9.

EPR Property Calculations

EPR property calculations were performed on the optimized geometries using the ORCA 4.1.0 program at the DFT level of theory. The cartesian reference system was oriented as such that the NH_2-Cu(II)-O axis was aligned with the y-axis and the N-Cu(II)-N axis was oriented along x. The integration grid size was kept large (AngularGrid = 7 for all atoms and IntAcc = 7 for the Cu(II) ion) to ensure that the core density was correctly described. Solvation effects were

accounted for in the property calculations by implementing the conductor-like polarized continuum model (CPCM) with a dielectric constant of 80.0 and a refractive index of 1.33 (water). The hyperfine coupling calculations included the Fermi-contact, spin dipolar and spin orbit contributions.

A variety of basis set combinations and functionals were employed to ensure extracted trends were not functional specific or basis set limited (**Table S3**). The fraction of Hartree-Fock exchange was also altered in the hybrid functional, following several studies showing improvements in the EPR property calculations (22). Calculations were performed using non-relativistic and scalar relativistic (zeroth order regular approximation, ZORA) approximations.

The overall accuracy of the spin-Hamiltonian parameters determined by DFT showed to be limited (**Tables S4-S6**). However, the trends predicted between Models A and B showed to be consistent. All the calculations show a change in both *g* and Cu(II) hyperfine tensors from rhombic to axial, going from the ‘resting state’ to the ‘4-coordinate’ model. The best agreement of the experimentally derived spin-Hamiltonian parameters was achieved using scheme 2 (**Table S3**). This scheme employed the hybrid functional, B3LYP, with an adjusted fraction of Hartree-Fock exchange (38%). This scheme also utilises the IGLO-III basis set which has additional flexibility in the core region, making it more suited for EPR properties calculations with respect to the Def2-TZVP basis set.

Superoxide model geometry optimizations

DFT calculations were performed to compare the 5-coordinate and the 4-coordinate Cu-superoxide binding energy. Cu-superoxide cluster models were constructed based on models (A) and (B) to compare the Cu-superoxide bond strength in the presence and absence of substrate. A five-coordinate superoxide model (D) was constructed, based off the resting state 5-coordinate model (A), replacing a water molecule with an end-on superoxide (retaining the water molecule H-bonded to the glutamate). The 4-coordinate superoxide model (E) was based on the 4-coordinate ‘substrate-bound’ model (B), replacing the water molecule with an end-on superoxide. Cluster models were also constructed for the resulting geometries following superoxide release. For the 5-coordinate superoxide model (D), this is simply the 4-coordinate water model (B) and superoxide. For the 4-coordinate superoxide model (E), an additional 3-coordinate Cu^{II} model (F) was generated to represent the geometry following superoxide release. A single superoxide molecule, model (G), was also produced as the remaining product following superoxide dissociation. All amino acid residues were retained in keeping with the previous

models. Geometry optimizations for models (D) and (E) were performed on the triplet potential energy surface. The individual models following superoxide dissociation (B), (F) and (G) were all optimized on the doublet potential energy surface. Both oxygen atoms on the superoxide molecules were treated using Ahlrich's Def2-TZVP basis set. All geometry optimizations were performed using the same basis set and functional schemes as outlined in the ***Geometry optimization*** section.

The atomic coordinates of all models used in the calculations are provided in the Appendix.

Superoxide bond strength calculations

To evaluate the superoxide binding energy, single point calculations were performed (as implemented by ORCA 4.2.0) on the optimized geometries: models (B), (D), (E), (F) and (G). These were completed using the hybrid B3LYP functional and the Def2-TZVP basis set across all atoms for improved accuracy. The RIJCOSX approximation and def2/J auxiliary basis set were implemented to help reduce the computation expense of these calculations. Grimme's dispersion correction with Becke-Johnson damping (D3BJ) was included. The conductor-like polarizable continuum model (CPCM) was implemented with a dielectric constant of 80.4 and refractive index of 1.33 to account for solvation effects in water. The relative binding energy of the superoxide in models (D) and (E) was examined by subtracting the electronic energies of superoxide (G) and the resulting geometry (models (B) and (F), respectively). The results are collected in **Table S10**. The strength of the Cu(II)-superoxide bond is shown to be 8.2 kcal mol^{-1} stronger in the 4-coordinate superoxide model (E) when compared with the 5-coordinate superoxide model (D).

In addition, differences in the Cu(II)-superoxide bonding are evident when assessing the Löwdin spin population analyses of the two superoxide models (**Table S9**). These values were obtained from the same single point frequency calculation described above. The spin population on the copper ion is shown to decrease by 12.5% upon changing from 5 to 4 coordinate (mimicking substrate binding). This decrease in spin population on the metal is in accord with an analogous increase of the spin population located on the two oxygen atoms. Therefore, this analysis shows that the decrease in coordination number from 5 to 4 leads to a large increase in covalency of the Cu(II)-superoxide bond. A reduction in spin population of $d(z^2)$ character (ca. 1.3%) is also seen following a reduction in coordination number.

Supplementary Discussion – Calculations used in EPR analysis

The Cu d based molecular orbitals are written as:

$$\psi_{x^2-y^2} = \alpha_{\mathrm{GS}}\left(a\, d_{x^2-y^2} - b\, d_{z^2}\right) - \sqrt{1-\alpha_{\mathrm{GS}}^2}\ \psi_L$$

$$\psi_{z^2} = \alpha_{\mathrm{GS}}\left(b\, d_{x^2-y^2} + a\, d_{z^2}\right) - \sqrt{1-\alpha_{\mathrm{GS}}^2}\ \psi_L$$

$$\psi_{xy} = \alpha_{\mathrm{xy}} d_{\mathrm{xy}} - \sqrt{1-\alpha_{\mathrm{xy}}^2}\ \psi_L$$

$$\psi_{xz} = \alpha_{\mathrm{xz}} d_{\mathrm{xz}} - \sqrt{1-\alpha_{\mathrm{xz}}^2}\ \psi_L$$

$$\psi_{yz} = \alpha_{\mathrm{yz}} d_{\mathrm{yz}} - \sqrt{1-\alpha_{\mathrm{yz}}^2}\ \psi_L$$

The $\psi_{x^2-y^2}$orbital is the SOMO; the α_i represents the metal d-orbital contribution to the molecular orbital, while a and b are the coefficient for the $d_{x^2-y^2}$ and d_{z^2} orbitals in the ground state orbital (GS), with $a^2 + b^2 = 1$.

The g values can be expressed as:

$$\Delta g_z \approx \frac{8\zeta_{\mathrm{Cu}}\, \alpha_{GS}^2\, \gamma_{xy}^2\, a^2}{\Delta E_{xy\rightarrow x^2-y^2}}$$

$$\Delta g_y \approx \frac{2\zeta_{\mathrm{Cu}}\, \alpha_{GS}^2\, \alpha_{xz}^2\, (a+\sqrt{3}b)^2}{\Delta E_{xz\rightarrow x^2-y^2}}$$

$$\Delta g_x \approx \frac{2\zeta_{\mathrm{Cu}}\, \alpha_{GS}^2\, \alpha_{yz}^2\, (a-\sqrt{3}b)^2}{\Delta E_{yz\rightarrow x^2-y^2}}$$

ζ_{Cu} represent the one-electron quasi-atomic copper spin–orbit coupling constant (usually taken as –830 cm^{-1}), and the **Δ***E* values are excitation energies of the ligand field transitions.

Similarly, the Cu hyperfine coupling can be written as:

$$A_z = P_{\mathrm{d}}\left[-K - \frac{4}{7}\alpha_{\mathrm{GS}}^2(a^2-b^2) + \Delta g_z + \frac{\Delta g_y\left(3a-\sqrt{3}b\right)}{14(a-\sqrt{3}b)} + \frac{\Delta g_x\left(3a+\sqrt{3}b\right)}{14(a-\sqrt{3}b)}\right]$$

$$A_y = P_{\mathrm{d}}\left[-K + \frac{2}{7}\alpha_{\mathrm{GS}}^2(a^2-b^2) - \frac{4\sqrt{3}}{7}\alpha_{\mathrm{GS}}^2(ab) + \Delta g_y - \frac{\Delta g_x\left(3a+\sqrt{3}b\right)}{14(a-\sqrt{3}b)}\right]$$

$$A_x = P_d\left[-K + \frac{2}{7}\alpha_{GS}^2(a^2 - b^2) + \frac{4\sqrt{3}}{7}\alpha_{GS}^2(ab) + \Delta g_x - \frac{\Delta g_y(3a-\sqrt{3}b)}{14(a-\sqrt{3}b)}\right]$$

$P_d = g_e g_{Cu} \mu_e \mu_{Cu}$ is the quasi atomic parameter usually taken as 1180 MHz, the term $-P_d K$ (in blue) represents the isotropic Fermi contact (A^{Fermi}), indicated in blue; the Spin-Dipolar (A^{SD}) contribution is in green and the Spin-Orbit contribution is indicated in red (A^{SO}).

The different Cu hyperfine contributions, the % d_{z^2} in the SOMO and the α_{GS}^2 were determined as follows:

1) The ratio between a/b (and therefore the % d_{z^2} in the ground state) was estimated from the rhombicity parameter (R_g) and the g values equations following Gewirth *et al.*:(23)

$$R_g = \frac{2(\Delta g_2 - \Delta g_1)}{\Delta g_2 + \Delta g_1} \approx 2\frac{(a+\sqrt{3}b)^2 - (a-\sqrt{3}b)^2}{(a+\sqrt{3}b)^2 + (a-\sqrt{3}b)^2}$$

   Assuming $\alpha_{yz}^2 \approx \alpha_{xz}^2$ and $\Delta E_{yz\rightarrow x^2-y^2} \approx \Delta E_{xz\rightarrow x^2-y^2}$.

2) A^{SO} was obtained from the experimental Δg and the a and b values obtained in 1)

3) A^{SO} was subtracted from A^{Total} to get $A^{Fermi} + A^{SD}$

4) The x, y and z components of $A^{Fermi} + A^{SD}$ were averaged to obtain A^{Fermi}

5) $A^{Fermi} + A^{SO}$ was subtracted from A^{Total} to get A^{SD}

6) α_{GS}^2 was calculated from A^{SD}

Supplementary Discussion - DFT calculations in presence of substrate

In searching for the origin of the change in EPR parameters and hydrogen-bonding patterns around the histidine brace upon substrate addition, we undertook a series of DFT calculations in which the effects of adding a chitin substrate to the Cu(II) active site were evaluated. To this end, three different cluster models of the enzyme Cu site were built, starting from the crystallographic coordinates of a spectroscopically similar AA10, *Ba*AA10, PDB:5IJU (20). In these models the protein backbone was truncated to only include important residue side chains near the metal, in order to reduce computational cost while still representing the major interactions imposed by the protein on the Cu(II) ion. All geometries were optimized with the uBP86 functional (see Methods

for details). Model A represents the Cu(II) resting state of the enzyme, where the metal first coordination sphere consisted of the His-brace and two water molecules in a distorted square-pyramidal geometric structure (**Figure S7-A**). The optimized geometry showed minimal distortion from the crystallographic coordinates (**Figure S8**), showing that the model faithfully represented the Cu(II) active site.

The spin-Hamiltonian parameters obtained from the EPR of *Bl*LPMO10A after addition of substrate suggested that the copper coordination sphere is near axial and, in accordance with previous studies, it is reasonable to think that water molecule in the nominal axial position is displaced by binding of substrate. Hence, a second model (Model B) was produced with a square planar-like symmetry about the copper center, achieved by the removal of the pseudo-axial water molecule from the optimized geometry structure of Model A (**Figure S7-B and S8-B**). Additionally, a third model was constructed based on Model B, but also including a short chitin oligosaccharide (NAG_2) bound to the active site (**Figure S9**). The positioning of this substrate molecule was informed from a recent combined molecular mechanics/quantum mechanics (QM/MM) study by Bissaro *et al.* (21) . Again, the calculated optimized geometry for these models showed good agreement with the crystallographic coordinates of the *Ba*AA10 resting state.

The optimized geometries were then used to calculate the EPR spin-Hamiltonian parameters for the models at the DFT level of theory, testing various functionals and basis sets to monitor the reproducibility of the trends (**Tables S3-S6**). Notwithstanding some deviation from experimental values, the results reproduced the experimental trends (**Table S7**) upon substrate addition. Most notably, the 'resting state' Model A predicts the rhombic *g* and A values seen experimentally, including the large A_1 value, that is a characteristic feature of chitin active AA10s. As expected, Model B afforded an almost axial set of spin-Hamiltonian parameters, commensurate with the values of *Bl*LPMO10A upon addition of substrate. These data confirm the conclusions from the LFT analysis above that most changes to the spectral envelope of *Bl*LPMO10A upon substrate binding are associated with a change in coordination number from 5 to 4, generating an almost square-planar coordination geometry for the Cu(II) ion.

The change of coordination number from 5 to 4 (between models A and B) emulates the changes seen in copper hyperfine values upon substrate binding. However, the decrease of a_{iso} observed in the HYSCORE spectra, assigned to the remote nitrogen of His121, is not reproduced in the differences between Models A and B. In Model C, the short substrate molecule introduces a hydrogen bond between the substrate and the N_δ-H group of His121. The calculated spin-

Hamiltonian parameters for this model show (**Table S8**) a decrease in the a_{iso} value from 1.92 to 1.48 MHz for the remote N of His121, with a simultaneous increase of the value of the electric field gradient asymmetry parameter, η, from 0.2 to 0.9, wholly in accord with the experimental data. In other words, the formation of a hydrogen bond between the N-H group of His121 and the substrate is required to fully reproduce the experimental trend.

Table S1. Input data for calculation of the structure of *apo-Bl*LPMO10A and structural assessment statistics for *apo-Bl*LPMO10A and Cu(I)-bound *Bl*LPMO10A.

	*apo-Bl*LPMO10A	Cu(I)-*Bl*LPMO10A
Total number of NOE distance constraints	1,623	1,209
Intraresidue	724	440
Sequential	496	421
Medium-range	101	98
Long-range	302	250
Torsion angle restraints[a]	264	264
Structure statistics (20 conformers)[b]		
CYANA target function value ($Å^2$)	3.69 ± 0.47	-
Maximum residual distance constraint violation (Å)	0.43 ± 0.15	-
Maximum torsion angle constraint violation (°)	4.20 ± 2.10	-
Ramachandran plot analysis[c]		
Residues in favored regions (%)	89.5	83.9
Residues in additionally allowed regions (%)	8.7	14.5
Residues in generously allowed regions (%)	0.6	0.7
Residues in forbidden regions (%)	1.2	0.9
rmsd to the lowest target energy conformer (Å)		
N, C^{α}, C’	2.41 ± 0.36	3.96 ± 1.15
Heavy atoms	2.71 ± 0.33	4.34 ± 1.15
N, C^{α}, C’ (Secondary structure)[d]	1.48 ± 0.23	2.52 ± 0.71
Heavy atoms (Secondary structure)[d]	1.82 ± 0.25	2.97 ± 0.79

[a] Calculated from secondary chemical shifts using the TALOS-N software (8).
[b] The values are the average and standard deviation over the 20 lowest CYANA target function values before energy minimization.
[c] Calculated using PROCHECK-NMR (24) and the PSVS server (25).
[d] Residues 33-36, 37-40, 41-47, 57-61, 82-84, 89-94, 103-107, 110-117, 125-132, 150-154, 159-161, 164-168, 175-184, 185-190 and 191-201.

Table S2. Davies ^{14}N ENDOR simulation parameters for *Bl*LPMO10A. The spectra and simulations are shown in Figure S5. The numbers in brackets represent the error on the measurement estimated from the quality of simulated fits. The Euler angles define the *zy'z''* rotations with respect to the *g* matrix.

	^{63}Cu-*Bl*LPMO10A	
	A **(MHz)**	**A Frame Euler angles**
N(Im)	33 33 40; 33 33 40 (±2)	[0 90 0]
NH_2	18 18 23 (±1)	[0 90 90]

Table S3. The functional and basis set schemes used to calculate the EPR properties of *Bl*LPMO10A.

Scheme	Functional	Cu(II)	Ligands[a]	Remaining atoms
**1**	B3LYP	CP(PPP)	IGLO III	Def2-SVP
**2**	B3LYP 38%HFX[b]	CP(PPP)	IGLO III	Def2-SVP
**3**	B3LYP 38%HFX[b] ZORA	CP(PPP)	ZORA-Def2-TZVP	ZORA-Def2-SVP
**4**	PBE0 ZORA	CP(PPP)	ZORA-Def2-TZVP	ZORA-Def2-SVP

a – 'Ligands' refers to the amino terminus nitrogen, all histidine ring atoms and the coordinating oxygen atoms.
b – '38%HFX' refers to an adjusted degree of Hartree-Fock exchange to 38%, from the default 20% in the B3LYP functional.

Table S4. Calculated spin Hamiltonian parameters for the 'resting state' Model A of *Bl*LPMO10A under different functional and basis set schemes.

	***g*-values**			**Cu(II) Hyperfine (MHz)**			**Principal N Super-Hyperfine (MHz)**[b]		
**Scheme**[a]	g_1	g_2	g_3	A_1	A_2	A_3	N1[c]	N2[c]	N3[c]
**1**	2.028	2.076	2.161	284	65.9	-374	56.9	44.0	44.0
**2**	2.036	2.104	2.220	309	50.7	-411	48.0	38.4	38.4
**3**	2.036	2.104	2.221	291	33.9	-432	46.6	35.0	35.7
**4**	2.032	2.086	2.183	251	21.8	-429	51.8	38.6	39.2

a – The scheme refers to the basis set and functional used in the calculation, as outlined in Table S3.
b – 'Principal N Superhyperfine' values refer to the largest individual value calculated in the tensor. The principal SHF value is a_{yy} N1 and a_{xx} for N2 and N3.
c – N1 refers to the amino terminus nitrogen, N2 refers to the coordinating ring nitrogen of His1 and N3 refers to the coordinating ring nitrogen of His121.

Table S5. Calculated spin Hamiltonian parameters for the '4-coordinate' Model B model of *Bl*LPMO10A under different functional and basis set schemes.

	***g*-values**			**Cu(II) Hyperfine (MHz)**			**Principal N Super-Hyperfine (MHz)[b]**		
**Scheme[a]**	g_1	g_2	g_3	A_1	A_2	A_3	N1[c]	N2[c]	N3[c]
**1**	2.041	2.043	2.154	3.8	-4.7	-564	48.5	45.2	47.1
**2**	2.055	2.060	2.213	22.7	-11.2	-603	42.4	39.8	41.9
**3**	2.057	2.060	2.214	-24.1	-55.1	-650	41.1	37.7	39.9
**4**	2.048	2.049	2.176	-53	-74.1	-638	44.4	41.3	43.2

a – The scheme refers to the basis set and functional used in the calculation, as outlined in Table S3.
b – 'Principal N Superhyperfine' values refer to the largest individual value calculated in the tensor. The principal SHF value is a_{yy} N1 and a_{xx} for N2 and N3.
c – N1 refers to the amino terminus nitrogen, N2 refers to the coordinating ring nitrogen of His1 and N3 refers to the coordinating ring nitrogen of His121.

Table S6. Calculated spin Hamiltonian parameters for 'substrate bound' Model C of *Bl*LPMO10A.

	***g*-values**			**Cu(II) Hyperfine (MHz)**			**Principal N Super-Hyperfine (MHz)[b]**		
**Scheme[a]**	g_1	g_2	g_3	A_1	A_2	A_3	N1[c]	N2[c]	N3[c]
**2**[d]	2.054	2.057	2.203	-8.97	-28.9	-638	43.1	42.7	45.4

a – The scheme refers to the basis set and functional used in the calculation, as outlined in Table S3.
b – 'Principal N Superhyperfine' values refer to the largest individual value calculated in the tensor. The principal SHF value is a_{yy} N1 and a_{xx} for N2 and N3.
c – N1 refers to the amino terminus nitrogen, N2 refers to the coordinating ring nitrogen of His1 and N3 refers to the coordinating ring nitrogen of His121.
d – Only scheme 2 was tested, due to size limitations of the model.

Table S7. EPR properties of *Bl*LPMO10A with and without β-chitin, determined experimentally and by DFT calculations (using B3LYP with 38% Hartree-Fock exchange). The signs –/+ refer to absence or presence of substrate, respectively.

	g_1	g_2	g_3	A_1 (MHz)	A_2 (MHz)	A_3 (MHz)
**EPR –**[a]	2.027	2.095	2.261	255	110	336[a]
**DFT –**[b]	2.036	2.104	2.220	309	50.7	–411
**EPR +**[a]	2.042	2.053	2.205	80	85	620[a]
**DFT +**[c]	2.055	2.060	2.213	22.7	–11.2	–603
**DFT +**[d]	2.054	2.057	2.203	–8.97	–28.9	–638

a – Signs of Cu hyperfine could not be determined from simulation of the CW EPR spectra.
b – Refers to the DFT 'resting state' Model A.
c – Refers to the DFT '4-coordinate' Model B.
d – Refers to the DFT 'substrate bound' Model C.

Table S8. EPR properties of the remote nitrogen atoms of the histidine rings of *Bl*LPMO10A with and without β-chitin, determined experimentally and by DFT calculations. The signs –/+ refer to absence or presence of substrate, respectively.

**System**	a_{iso} His32 **(MHz)**	T His32 **(MHz)**	η His32	a_{iso} His121 **(MHz)**	T His121 **(MHz)**	η His121
**EPR –**	1.3	0.25	0.85	1.6	0.3	0.7
**DFT –**[a]	1.21	0.24	0.5	1.75	0.21	0.2
**EPR+**	1.25	0.30	0.8	1.55	0.35	0.9
**DFT +**[b]	1.13	0.21	0.5	1.92	0.21	0.2
**DFT +**[c]	1.38	0.31	0.7	1.48	0.23	0.9

a – Refers to the DFT 'resting state' Model A.
b – Refers to the DFT '4-coordinate' Model B.
c – Refers to the DFT 'substrate bound' Model C.

Table S9. Löwdin spin population analysis of 4-coordinate and 5-coordinate superoxide models (adding up to 200% for a total of 2 spins).

Model	N (NH_2)	N (His1)	N (His2)	Cu[a]	O (O_2 proximal)	O (O_2 distal)	O (Water)	O-O distance / Å
**4 - coordinate (E)**	5.5	3.8	3.3	Total: **41.0**<br>s = -0.9<br>p = -1.3<br>d = 43.2<br>(z2 = 0.2)<br>(xz = 0.2)<br>(yz = 0.8)<br>(xy = 0.7)<br>(x_2-y_2 = 41.2)	71.6	72.5	-	1.28
**5 - coordinate (D)**	6.8	5.4	4.8	Total: **53.5**<br>s = -0.5<br>p = -1.4<br>d = 55.4<br>(z2 = 1.5)<br>(xz = 0.6)<br>(yz = 1.3)<br>(xy = 0.3)<br>(x_2-y_2 = 51.7)	64.9	60.9	0.5	1.30

Table S10. Calculated energies of superoxide cluster models (D) and (E) and the resulting geometries following superoxide dissociation (B), (F) and (G). Cu(II)-superoxide binding energy calculated by the difference in electronic energy.

Model	5-coordinate superoxide (D)	4-coordinate superoxide (E)	4-coordinate water (B)	3-coordinate Hisbrace (F)	Superoxide (G)
**Single point energy / Hartree**	-4489.6	-4413.2	-4339.1	-4262.7	-150.5
**Cu(II)-O_2 binding energy / Hartree**	-0.0417	-0.0549	-	-	-
**Cu(II)-O_2 binding energy / kcal mol^{-1}**	-26.2	-34.4	-	-	-

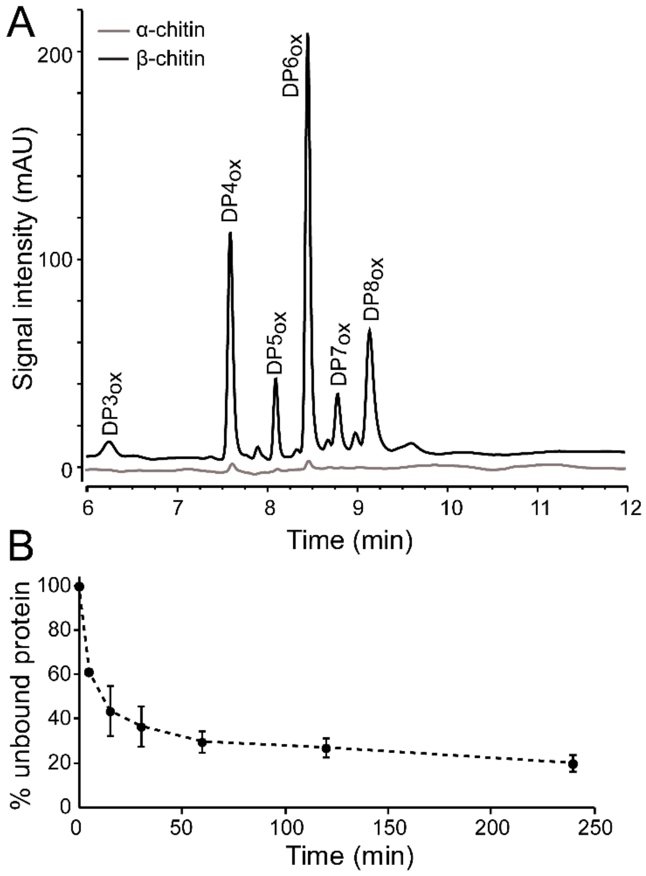

Figure S1. *Bl*LPMO10A activity and binding towards chitin. A) Chromatographic analysis of C1-oxidized chito-oligosaccharides obtained from degradation reactions containing 10 mg/mL α-chitin (grey) or β-chitin (black), 1 µM Cu(II)-loaded *Bl*LPMO10A, 50 mM Tris/HCl pH 8.0 and 2 mM ascorbic acid. Reactions were incubated for 24 h in an Eppendorf Thermomixer set to 40 °C and 800 rpm. B) Binding of *Bl*LPMO10A to 10 g/L β-chitin. The percentage of free protein was determined by measuring the reduction in concentration of soluble protein over time. The binding experiment was carried out at 40 °C using in 50 mM sodium phosphate buffer pH 7.0. The error bars show ± S.D. (n = 3).

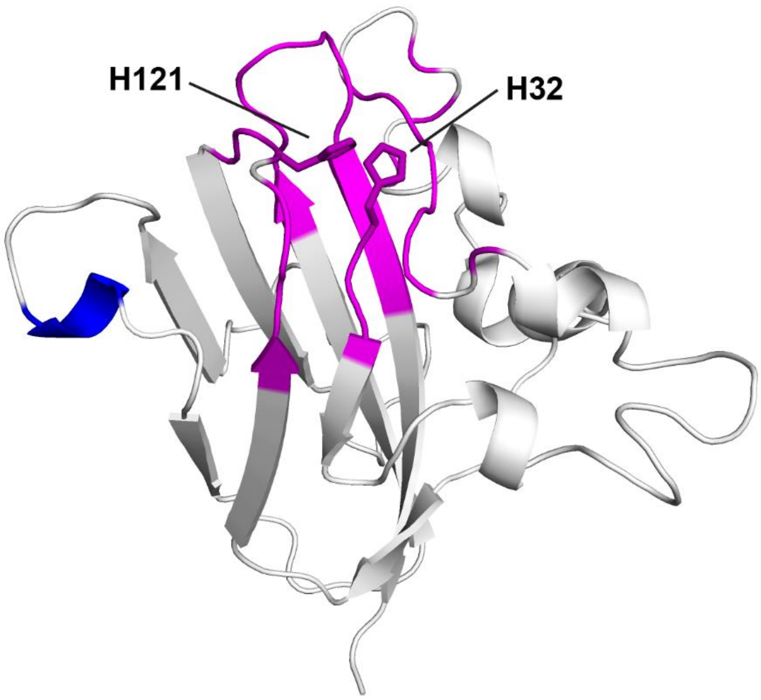

Figure S2. Effect of Cu(II)-binding on *apo-Bl*LPMO10A. Residues with less than 30% remaining intensity (see Figure 1E) and within a 12 Å radius from the expected Cu(II) coordination site are colored pink, whereas residues with less than 30% remaining intensity and further than 12 Å from the Cu(II) site (Ala160-Arg162) are colored blue. The side-chains of His32 and His121 are shown as sticks.

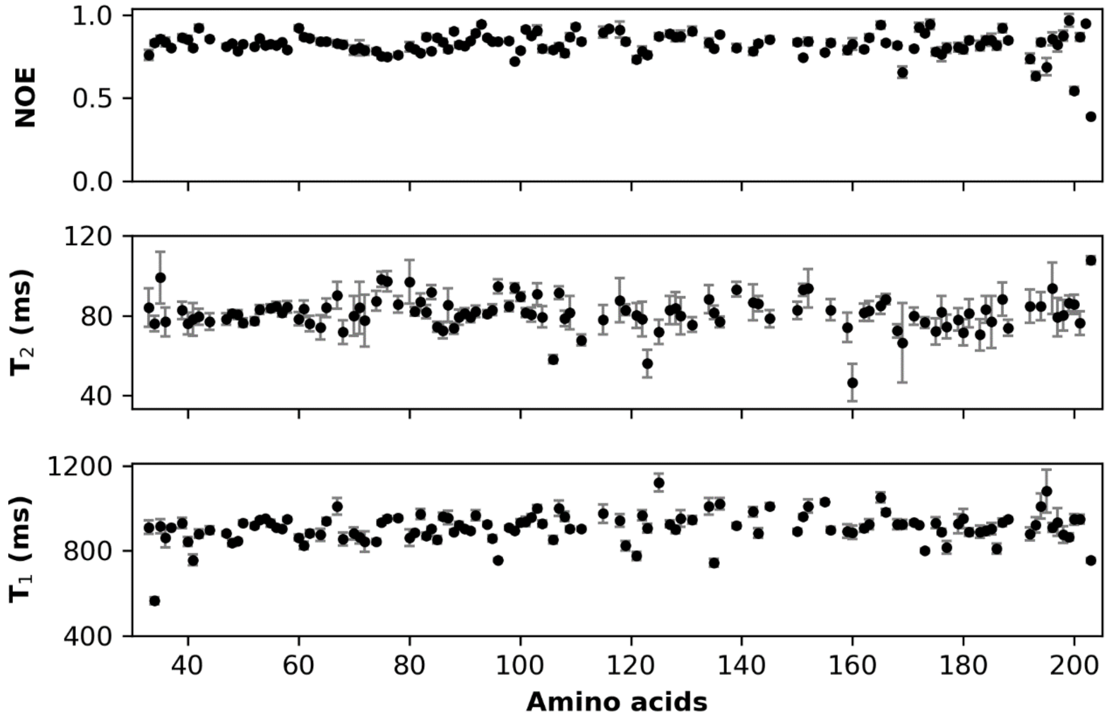

Figure S3. Backbone dynamics of *apo-Bl*LPMO10A. Decreased {[1]H}-^{15}N NOE and increased ^{15}N-T_2 values are an indication of conformational flexibility. The data show that *apo-Bl*LPMO10A has an overall rigid backbone, with some flexibility in loops (e.g. between α2 and β2), a flexible N-terminus and a flexible C-terminus. The rotational correlation time, calculated from the average T_1/T_2 ratio, is $\tau_c = 10.2 \pm 0.9$ ns.

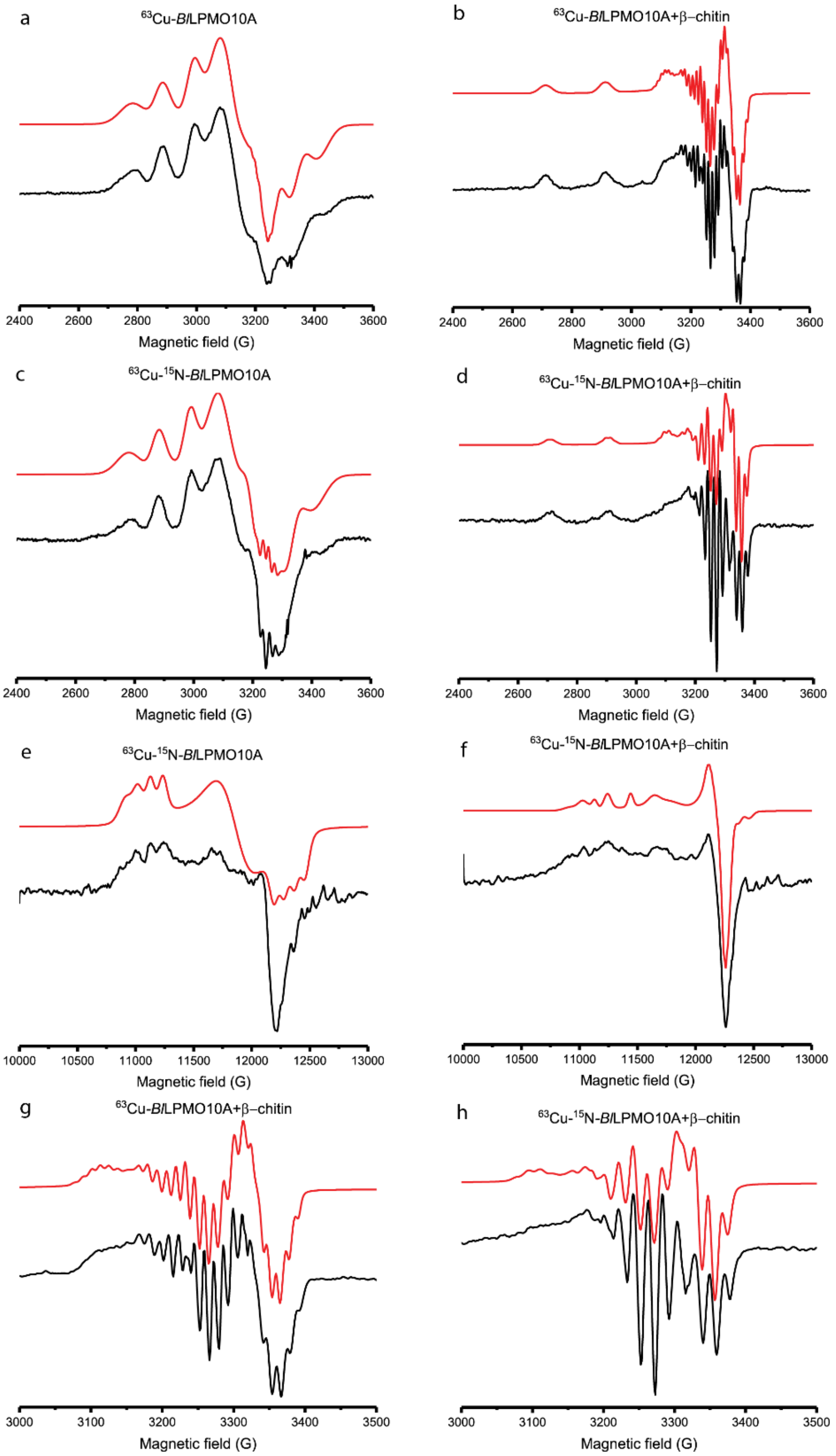

Figure S4. **X and Q band CW-EPR spectra.** The panels show X-band (**a-d**, **g-h**) and Q-band (**e-f**) CW-EPR spectra (in black, bottom lines) and corresponding simulations (in red, top lines) for ^{63}Cu–*Bl*LPMO10A (**a**), ^{63}Cu–^{15}N–*Bl*LPMO10A (**c**, **e**) ^{63}Cu–*Bl*LPMO10A with squid pen β-chitin (**b**) and ^{63}Cu–^{15}N–*Bl*LPMO10A with squid pen β-chitin (**d**, **f**). **g** and **h**: detail of the superhyperfine (SHF) coupling with simulations for ^{63}Cu–*Bl*LPMO10A and ^{63}Cu–^{15}N-*Bl*LPMO10A, respectively, after addition of β-chitin. The spectra shown in panels **b** and **d** were obtained by subtraction of the spectrum of ^{63}Cu–*Bl*LPMO10A and ^{63}Cu–^{15}N–*Bl*LPMO10A, respectively, to correct for the free enzyme not bound to the substrate.

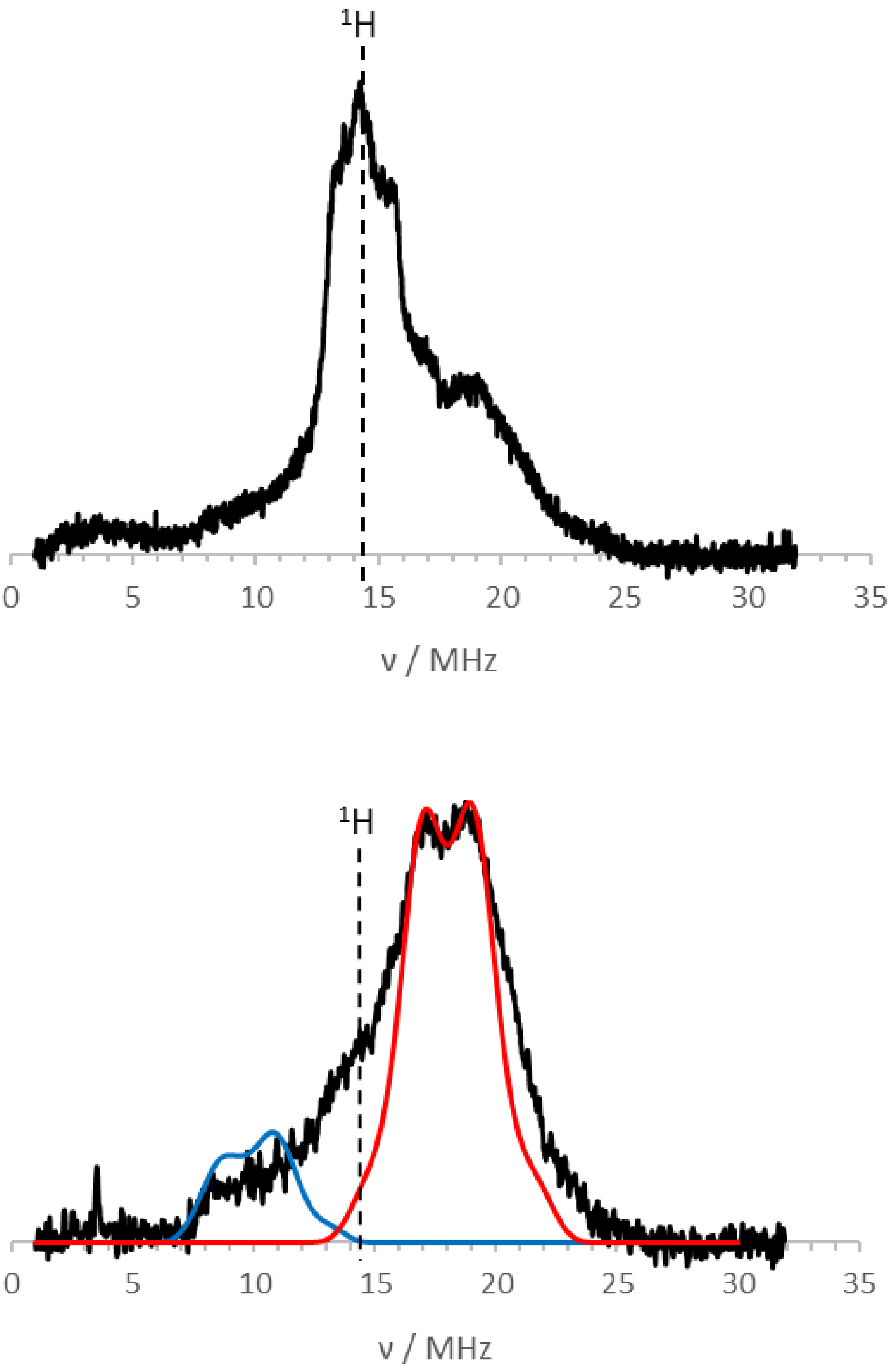

Figure S5. **Davies ^{14}N ENDOR spectra of ^{63}Cu-*Bl*LPMO10A recorded at 3400 G near $g_{\perp}$. Top**: spectrum recorded with soft pulses, π/2 = 128 ns. **Bottom**: spectrum recorded with hard pulses, π/2 = 32 ns, with simulations for the coordinated nitrogen atoms in red (His N) and blue (N-terminus). The dashed line indicates the position of the ^{1}H Larmor frequency at this field.

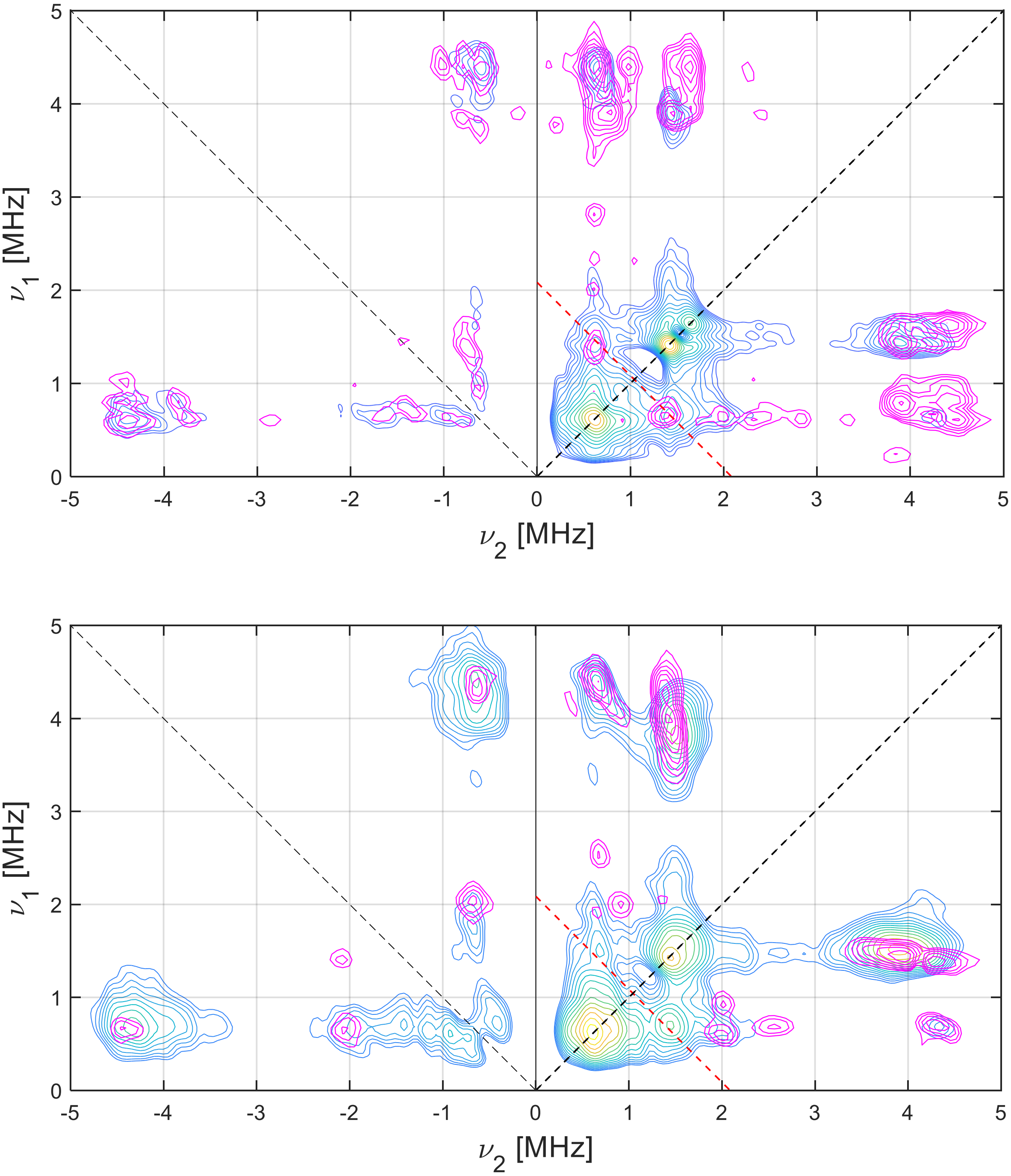

Figure S6. ^{14}N HYSCORE spectra and simulations (in pink) of ^{63}Cu-*Bl*LPMO10A (top) and ^{63}Cu-*Bl*LPMO10A with squid pen β-chitin (bottom). **Top,** ^{63}Cu-*Bl*LPMO10A near $g_{\perp}$ with $\tau = 200$ ns at 3385 G and 32 ns increments. **Bottom,**^{63}Cu-*Bl*LPMO10A with squid pen β-chitin near $g_{\perp}$ with $\tau = 200$ ns at 3390 G and 32 ns increments.

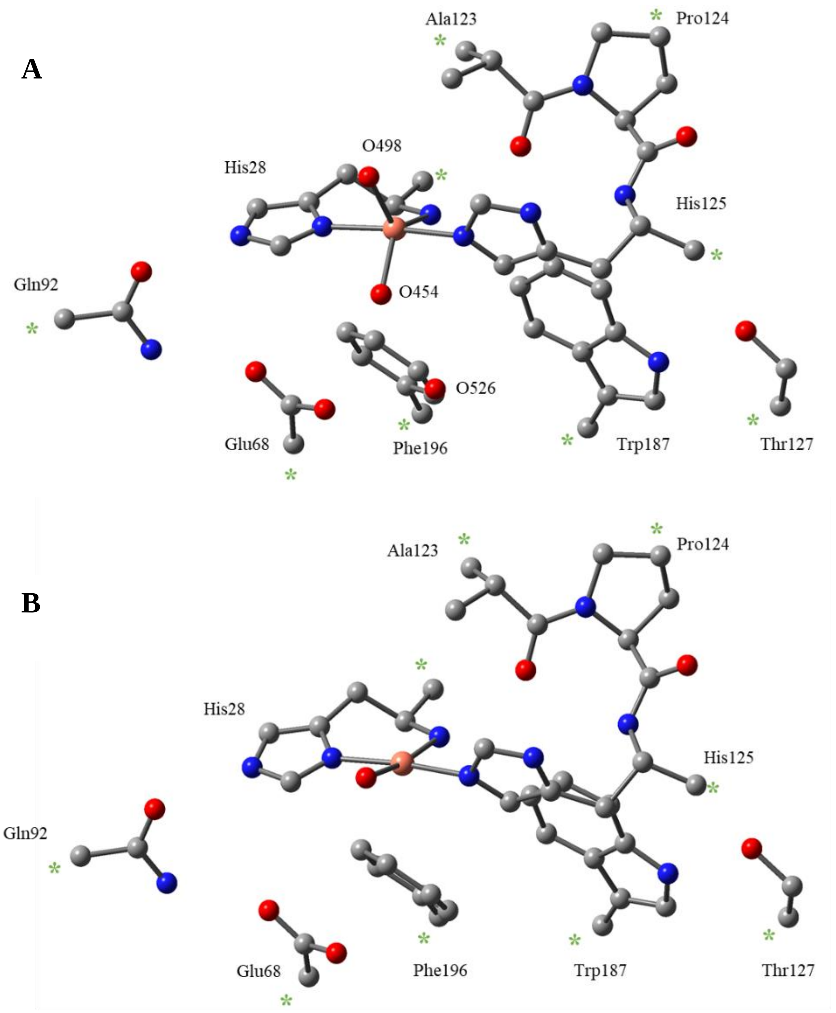

Figure S7. Starting structure for geometry optimization of Cu(II)-*Bl*LPMO10A. The panels show the structure in the 'resting state' (Model A) and in the '4-coordinate' state (Model B). Hydrogen atoms are omitted for clarity. Residue labels are given as found in the crystallographic coordinates of *Ba*AA10 (PDB:5IJU). Atoms that were kept frozen throughout the geometry optimizations are denoted with a green asterisk.

A

B

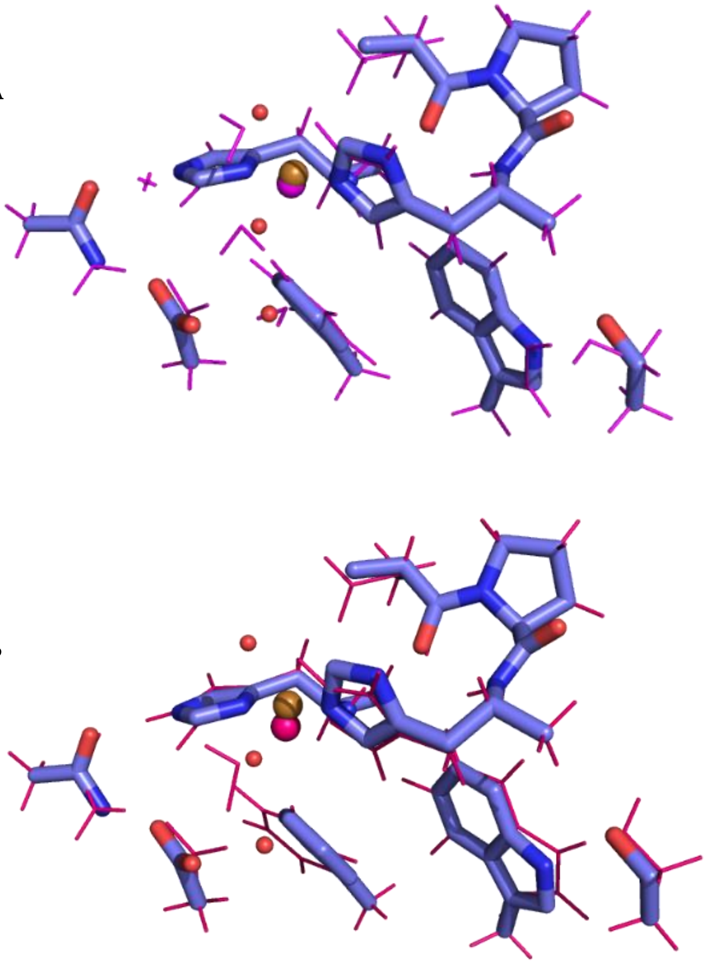

Figure S8. Geometry optimized structure (pink wires) of Cu(II)-*Bl*LPMO10A. The panels show the structure in the ‘resting state’ (Model A) and the ‘4-coordinate’ state (Model B), plotted against the crystallographic coordinates (PDB:5IJU) (blue sticks). The water molecules present in the crystal structures are shown as red spheres.

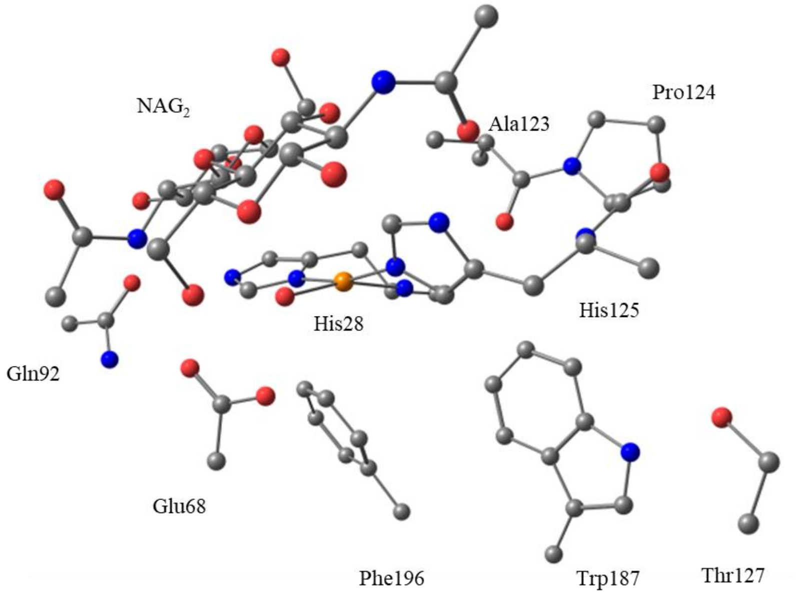

Figure S9. Geometry optimized structure of Cu(II)-*Bl*LPMO10A in the 'substrate-bound' case (Model C). The figure shows the structure with a chitin oligosaccharide bound to the active site, with hydrogen atoms omitted for clarity. Residue labels are given as found in the crystallographic coordinates of *Ba*AA10 (PDB:5IJU).

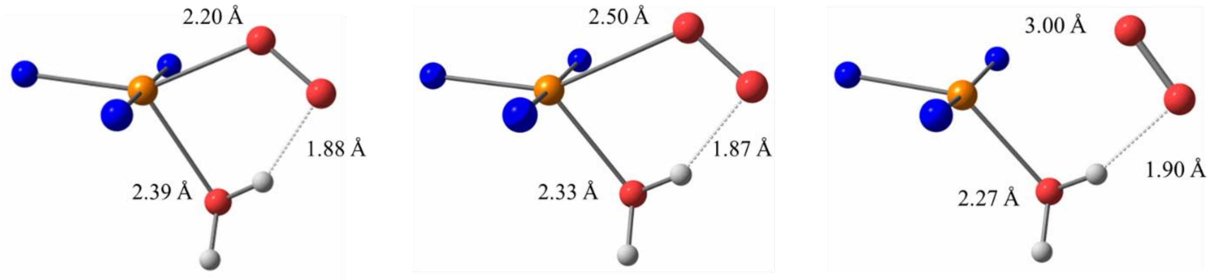

Figure S10. Superoxide release from Cu(II)-AA10. The pictures show DFT-optimized structures with Cu-O distances fixed at 2.20, 2.50 and 3.00 Å (Cu-O = 2.20 & O-O = 1.30, Cu-O = 2.50 & O-O = 1.29, and Cu-O = 3.00 & O-O = 1.28 Å, respectively). A relaxed surface scan was performed along the Cu-O coordinate between 2.20 and 3.00 Å and a barrier of 3.83 kcal mol^{-1} was determined. DFT optimizations were performed using the uBP86 functional and Ahlrichs def2-SVP basis set. The basis set size was increased to Ahlrichs def2-TZVP on the copper ion and all ligating atoms for improved accuracy.

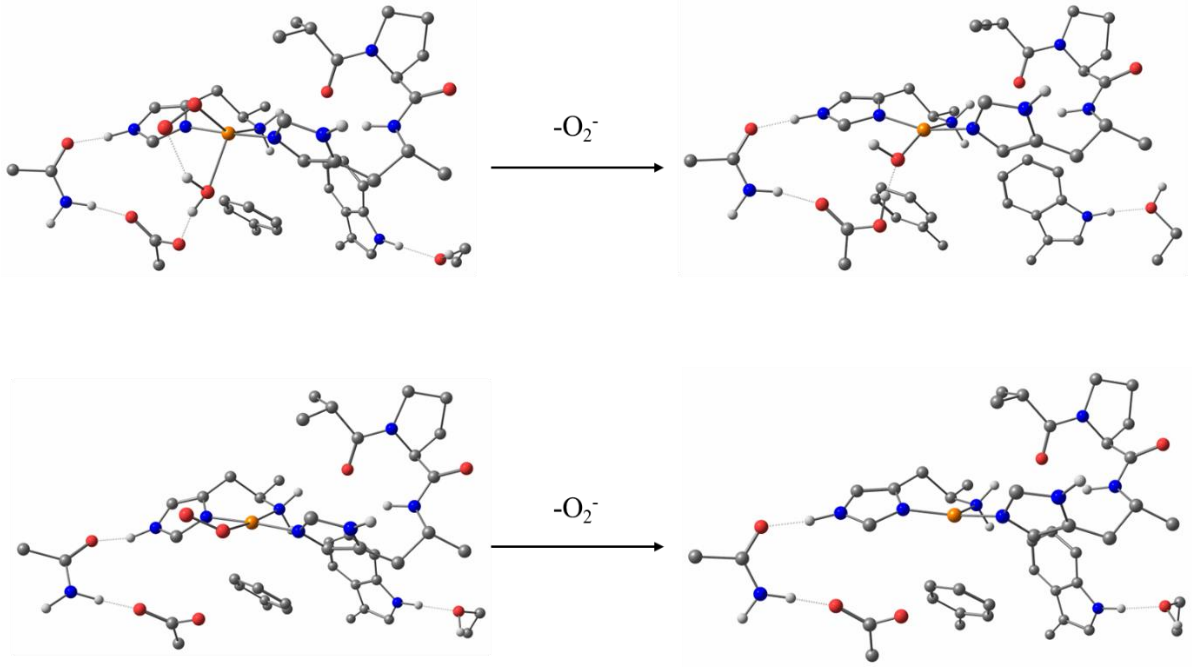

Figure S11. Top: DFT optimized geometries of the 5-coordinate superoxide model (D) (left) and resulting structure following superoxide removal (model (B)) (right). Bottom: DFT optimized geometries of the 4-coordinate superoxide model (E) (left) and resulting structure following superoxide removal (model (F)) (right). Hydrogen atoms attached to carbon atoms were omitted for clarity.

References

1. Z. Forsberg *et al.*, Comparative study of two chitin-active and two cellulose-active AA10-type lytic polysaccharide monooxygenases. *Biochemistry* **53,** 1647-1656 (2014).
2. J. S. M. Loose; Z. Forsberg; M. W. Fraaije; V. G. H. Eijsink; G. Vaaje-Kolstad, A rapid quantitative activity assay shows that the *Vibrio cholerae* colonization factor GbpA is an active lytic polysaccharide monooxygenase. *FEBS Lett.* **588,** 3435-3440 (2014).
3. G. Courtade *et al.*, ^{1}H, ^{13}C, ^{15}N resonance assignment of the chitin-active lytic polysaccharide monooxygenase *Bl*LPMO10A from *Bacillus licheniformis*. *Biomol. NMR Assign.* **9,** 207-210 (2015).
4. G. Courtade; S. B. Le; G. I. Sætrom; T. Brautaset; F. L. Aachmann, A novel expression system for lytic polysaccharide monooxygenases. *Carbohydr. Res.* **448,** 212-219 (2017).
5. R. Keller, The computer aided resonance assignment tutorial. CANTINA verlag Goldau: (2004).
6. N. A. Farrow *et al.*, Backbone Dynamics of a Free and a Phosphopeptide-Complexed Src Homology 2 Domain Studied by ^{15}N NMR Relaxation. *Biochemistry* **33,** 5984-6003 (1994).
7. L. E. Kay; D. A. Torchia; A. Bax, Backbone dynamics of proteins as studied by ^{15}N inverse detected heteronuclear NMR spectroscopy: application to staphylococcal nuclease. *Biochemistry* **28,** 8972-8979 (1989).
8. Y. Shen; A. Bax, Protein backbone and sidechain torsion angles predicted from NMR chemical shifts using artificial neural networks. *J. Biomol. NMR* **56,** 227-241 (2013).
9. I. Solomon, Relaxation Processes in a System of Two Spins. *Phys. Rev.* **99,** 559-565 (1955).
10. N. Bloembergen; L. O. Morgan, Proton Relaxation Times in Paramagnetic Solutions. Effects of Electron Spin Relaxation. *J. Chem. Phys.* **34,** 842-850 (1961).
11. G. Lipari; A. Szabo, Model-free approach to the interpretation of nuclear magnetic resonance relaxation in macromolecules. 1. Theory and range of validity. *J. Am. Chem. Soc.* **104,** 4546-4559 (1982).
12. G. M. Clore; J. Iwahara, Theory, Practice, and Applications of Paramagnetic Relaxation Enhancement for the Characterization of Transient Low-Population States of Biological Macromolecules and Their Complexes. *Chem. Rev.* **109,** 4108-4139 (2009).
13. P. Güntert; W. Braun; K. Wüthrich, Efficient computation of three-dimensional protein structures in solution from nuclear magnetic resonance data using the program DIANA and the supporting programs CALIBA, HABAS and GLOMSA. *J. Mol. Biol.* **217,** 517-530 (1991).
14. P. Güntert, Automated NMR Structure Calculation With CYANA. In *Protein NMR Techniques*, (Downing, A. K., Ed. Humana Press, 2004) pp 353-378.
15. E. Krieger; G. Koraimann; G. Vriend, Increasing the precision of comparative models with YASARA NOVA—a self-parameterizing force field. *Proteins: Struct., Funct., Bioinf.* **47,** 393-402 (2002).
16. U. Essmann *et al.*, A smooth particle mesh Ewald method. *J. Chem. Phys.* **103,** 8577-8593 (1995).
17. E. Krieger *et al.*, Improving physical realism, stereochemistry, and side-chain accuracy in homology modeling: Four approaches that performed well in CASP8. *Proteins: Struct., Funct., Bioinf.* **77,** 114-122 (2009).
18. B. Bissaro; I. Isaksen; G. Vaaje-Kolstad; V. G. H. Eijsink; Å. K. Røhr, How a lytic polysaccharide monooxygenase binds crystalline chitin. *Biochemistry* **57,** 1893-1906 (2018).
19. S. Stoll; A. Schweiger, EasySpin, a comprehensive software package for spectral simulation and analysis in EPR. *J. Magn. Reson.* **178,** 42-55 (2006).

20. R. C. Gregory *et al.*, Activity, stability and 3-D structure of the Cu(II) form of a chitin-active lytic polysaccharide monooxygenase from *Bacillus amyloliquefaciens*. *Dalton Trans.* **45,** 16904-16912 (2016).
21. B. Bissaro *et al.*, Molecular mechanism of the chitinolytic monocopper peroxygenase reaction. *bioRxiv* 541292 (2019).
22. F. Neese, Sum-over-states based multireference ab initio calculation of EPR spin Hamiltonian parameters for transition metal complexes. A case study. *Magn. Reson. Chem.* **42,** S187-S198 (2004).
23. A. A. Gewirth; S. L. Cohen; H. J. Schugar; E. I. Solomon, Spectroscopic and theoretical studies of the unusual EPR parameters of distorted tetrahedral cupric sites: correlations to x-ray spectral features of core levels. *Inorg. Chem.* **26,** 1133-1146 (1987).
24. Y. J. Huang; R. Powers; G. T. Montelione, Protein NMR Recall, Precision, and F-measure Scores (RPF Scores): Structure Quality Assessment Measures Based on Information Retrieval Statistics. *J. Am. Chem. Soc.* **127,** 1665-1674 (2005).
25. A. Bhattacharya; R. Tejero; G. T. Montelione, Evaluating protein structures determined by structural genomics consortia. *Proteins: Struct., Funct., Bioinf.* **66,** 778-795 (2007).

Appendix

The atomic coordinates of all models used in the calculations are provided below:

‘Resting State’ (A) Model

N	27.996835319	2.235009655	75.770343883
C	27.944105399	3.609911146	76.354848730
C	28.544005970	4.658001781	75.421027631
C	28.638223810	3.627780123	77.734230194
C	27.794872493	2.972032572	78.776840611
C	27.301476199	3.447222877	79.981148544
N	27.282407350	1.692906765	78.588885848
C	26.511153470	1.408549359	79.653348639
N	26.506664936	2.447226697	80.508902835
C	21.658000973	0.989999387	78.100003330
C	23.049361211	0.556868777	78.586503492
O	23.595135257	1.169223773	79.540287803
O	23.626026509	-0.404259373	77.947003899
C	23.453003487	2.661001950	84.412007330
C	23.942974047	2.438202458	82.993638733
N	23.024911952	2.197398520	82.041151964
O	25.180235752	2.480957582	82.778004296
C	32.303268129	1.781352508	75.219976261
C	31.574052249	1.561766863	73.885758488
O	30.316558933	1.538173464	73.840475732
C	31.604296475	0.997668197	76.343203127
N	32.325235441	1.421736779	72.757425590
C	31.702167490	1.379275321	71.411337842
C	31.264902455	-0.068612467	71.048943504
O	31.989011510	-0.822591005	70.385528804
C	32.830754498	1.872119197	70.488962318
C	34.105001619	1.306000412	71.147005340
C	33.810699110	1.462910457	72.658701224
N	30.029175628	-0.426875772	71.504223267
C	29.505346191	-1.789882703	71.315568402
C	29.291004058	-2.135003278	69.835999927
C	28.183529899	-1.951364056	72.097540502
C	28.280799980	-1.565444074	73.535224599
C	27.619687995	-0.592051846	74.263819119
N	29.169839719	-2.147667976	74.429650568
C	29.039985337	-1.549228077	75.638591978
N	28.099914602	-0.601481689	75.563521306
C	26.451343423	-0.457578590	66.197507869
C	25.238000476	0.404003522	65.840001012
O	26.555714005	-0.724962687	67.615159961
C	23.034996959	4.172974679	69.639919118
C	24.122077526	3.144716385	69.667984563
C	24.351121633	2.120555601	68.757062021
C	25.172968350	3.038983792	70.649254340
C	26.019546695	1.941650415	70.253896492
C	25.475966934	3.753109749	71.827424622
N	25.486404956	1.405218696	69.102493054
C	27.172341604	1.589898122	70.986030150
C	26.593909013	3.372128043	72.575537845
C	27.449702715	2.318464040	72.155016560
C	22.458001243	4.792021941	73.872079785
C	23.260307190	3.811523900	74.691299613
C	23.585018711	2.538830121	74.172588961
C	23.675044726	4.133385161	76.004732362
C	24.296563088	1.606245942	74.944562786
C	24.385968695	3.204254091	76.783383875
C	24.694286517	1.942102957	76.248794730
Cu	27.595691606	0.592236127	77.024357965
O	26.139480908	-0.910524902	77.693189323
O	28.721408198	-1.000187398	78.549293864
O	24.529902261	-1.515398813	75.668949818
H	21.078494047	0.129419674	77.712776290

H	21.810276225	1.704253627	77.261597651
H	23.844381816	1.842097188	85.049664328
H	22.350819989	2.696614346	84.507742374
H	22.037980242	2.149773218	82.299250230
H	23.272370156	1.920696372	81.058974119
H	32.129730589	1.152511360	77.306834368
H	31.588113970	-0.088921134	76.125583746
H	30.552213627	1.321562441	76.473805659
H	33.338087310	1.393282537	75.128536549
H	32.844074948	2.981871374	70.494204877
H	32.689682723	1.527147993	69.447277962
H	34.207752971	0.235305942	70.886041579
H	35.028920542	1.840205358	70.852684891
H	34.267754104	0.656097242	73.266347522
H	34.182390564	2.437030844	73.042798180
H	30.810602676	2.039776446	71.410561539
H	27.391027595	0.063633798	65.918951082
H	24.298660746	-0.059398835	66.208356003
H	25.328955832	1.421203045	66.268286385
H	25.156104053	0.508095084	64.737987803
H	25.959173974	-1.470958809	67.826130759
H	22.363762342	4.093830569	70.524084341
H	23.449064398	5.204883951	69.651834073
H	23.767807963	1.849142215	67.869132761
H	24.834993530	4.582596378	72.161980533
H	26.800786943	3.910027229	73.509419270
H	27.843169178	0.788147011	70.642296188
H	28.354957927	2.084720559	72.738461994
H	22.724647943	5.842355435	74.107705480
H	22.606236861	4.632986953	72.784840676
H	23.279866587	2.281784712	73.145715218
H	23.428514487	5.124360393	76.420715423
H	24.514228931	0.599437806	74.556358356
H	24.686203863	3.444627094	77.815347245
H	25.212160116	1.206100696	76.871975555
H	28.898705384	2.050336484	75.276539488
H	26.866357878	3.823935721	76.505713460
H	28.388600424	5.672517161	75.839707379
H	28.833517626	4.674797533	78.035181233
H	29.630562440	3.130863012	77.646073498
H	27.460651446	4.401960681	80.492452302
H	25.905551914	0.511182488	79.787211432
H	29.630930642	0.168523725	72.247030429
H	30.259277348	-2.503436852	71.725177523
H	28.945486028	-3.184993648	69.743964890
H	27.859321202	-3.009281316	72.000245973
H	27.392385040	-1.330993930	71.629333468
H	26.836468783	0.096323049	73.934016807
H	29.817670626	-2.912253154	74.220980633
H	29.607824858	-1.801668311	76.537773664
H	25.824860109	-1.350523947	76.841085892
H	27.780119931	-1.313458303	78.581621832
H	24.200149903	-2.431525913	75.587028947
H	28.808197712	-0.453119493	79.356936882
H	25.194791090	-0.610342408	78.020346807
H	25.931499379	2.472520613	81.421062064
H	25.876978142	0.601512577	68.565238973
H	22.402065990	4.073649808	68.735178425
H	21.369225932	4.679704530	74.070091032
H	23.887430491	3.606059726	84.794121459
H	21.099262507	1.506510498	78.903500773
H	26.432715008	-1.418761691	65.637243574

H	29.635599837	4.501274709	75.301064736
H	28.097244109	4.635885225	74.411631025
C	32.394500192	3.296120590	75.535725742
H	32.954502915	3.454137171	76.479192155
H	32.909016374	3.854718549	74.727330234
H	31.383445409	3.732361778	75.661350304
H	27.267294365	2.159704198	75.042927312
H	23.925669104	-1.097156362	76.350160220
H	28.522643402	-1.480038290	69.372504158
H	30.237891126	-2.027775557	69.275915551

‘4-coordinate’ (B) Model

N	27.928399365	2.266239500	75.791547487
C	27.962304440	3.634060574	76.391189978
C	28.543980676	4.657993335	75.421014402
C	28.725277013	3.584724252	77.732296580
C	27.890661831	2.956702773	78.802221539
C	27.500178393	3.445205029	80.039512032
N	27.244122381	1.739642948	78.604330944
C	26.489547851	1.508936543	79.693002536
N	26.626760972	2.517711959	80.575492384
C	21.657999703	0.990002350	78.100013913
C	23.048104044	0.469148668	78.434425359
O	23.603545471	0.702661115	79.523947339
O	23.613289053	-0.206788637	77.459657053
C	23.453018833	2.660996547	84.411965525
C	24.004202342	2.378308711	83.025399422
N	23.145422863	1.915939756	82.095425441
O	25.224019561	2.578399424	82.814166152
C	32.302372338	1.781967688	75.213292223
C	31.571297405	1.566831312	73.879203569
O	30.314697287	1.543256582	73.834098617
C	31.602459595	0.989671895	76.329793246
N	32.324235084	1.429835080	72.751214108
C	31.702282903	1.385078718	71.405252833
C	31.257433489	-0.062818298	71.050470709
O	31.985907568	-0.832057510	70.409964092
C	32.833530350	1.868528724	70.481123615
C	34.104965582	1.305998973	71.147028624
C	33.808984364	1.475001697	72.655648401
N	30.008492312	-0.398135217	71.486200019
C	29.457414957	-1.751663686	71.312242523
C	29.291031999	-2.134953673	69.836061311
C	28.097941776	-1.845732355	72.035252107
C	28.140279078	-1.470700634	73.478878334
C	27.457350491	-0.490419444	74.178696725
N	28.959808528	-2.081765004	74.418104450
C	28.764806130	-1.490274790	75.624998473
N	27.855006026	-0.521506908	75.503556527
C	26.585678402	-0.302019418	65.947056215
C	25.237995608	0.403996679	65.839987253
O	27.245265563	0.117756176	67.159114479
C	23.034999234	4.173019230	69.640112797
C	24.300263518	3.373936861	69.539015071
C	24.786494276	2.690272548	68.430446415
C	25.266099047	3.151931876	70.587896767
C	26.310592118	2.322028202	70.041194226
C	25.355423003	3.569515380	71.933331510
N	25.987189781	2.065523088	68.727050263
C	27.425592077	1.922724121	70.808730900
C	26.462368461	3.172371908	72.692594498
C	27.495298479	2.366812076	72.139552943
C	22.458023415	4.791982041	73.871910448
C	23.130536419	4.305695196	75.136877970
C	23.847012621	3.087890708	75.169666850
C	23.066228259	5.071086574	76.324798342
C	24.473303156	2.651747338	76.350148049
C	23.689122905	4.633746772	77.506927928
C	24.395705451	3.417438088	77.526255598
Cu	27.266161344	0.671299589	76.964825524
O	26.024733743	-0.711481261	77.652490830
H	21.105525532	0.272607153	77.463731718

H	21.787583834	1.929651813	77.520596047
H	23.963919253	1.991180088	85.133280154
H	22.358725845	2.520851895	84.500699025
H	22.167859601	1.757193715	82.344429796
H	23.427494815	1.618237029	81.140167901
H	32.119387873	1.145336987	77.298027800
H	31.594060388	-0.096974029	76.108629436
H	30.547436056	1.306800380	76.450973778
H	33.337197816	1.394489495	75.119678122
H	32.848500934	2.978335041	70.476790183
H	32.694591636	1.515114306	69.441865537
H	34.205802106	0.232847945	70.894979383
H	35.030729005	1.835332297	70.849327791
H	34.268017951	0.674590818	73.270274546
H	34.177606801	2.453332710	73.032480812
H	30.815272136	2.052027365	71.400605069
H	27.213682095	-0.060357758	65.060077886
H	24.618456634	0.208619097	66.739121313
H	25.375795957	1.498839460	65.729521502
H	24.682269960	0.035534919	64.954013302
H	28.210654784	0.028793430	67.047716317
H	22.353869902	3.763808115	70.418770660
H	23.235793107	5.229384387	69.924808569
H	24.345363549	2.609623411	67.430006662
H	24.572915534	4.201247206	72.374923045
H	26.519158282	3.515571919	73.736359050
H	28.219529245	1.291342541	70.379726904
H	28.372177461	2.100975075	72.751757573
H	23.058376221	5.588844342	73.380328068
H	22.320380074	3.974471545	73.136925054
H	23.913283499	2.470543046	74.259666665
H	22.509542048	6.023019300	76.322549339
H	24.995279255	1.682735550	76.361759256
H	23.615370466	5.242273520	78.422404039
H	24.859165024	3.053486360	78.453742974
H	28.844738988	2.023918363	75.357192450
H	26.904927967	3.893804115	76.607685387
H	28.521082488	5.671903792	75.868789902
H	28.993213689	4.612553853	78.043947054
H	29.682891085	3.036794051	77.587908981
H	27.772911902	4.367105898	80.563151970
H	25.767422893	0.694778131	79.806136398
H	29.606740310	0.212235867	72.214685528
H	30.170354668	-2.476306374	71.773408854
H	28.894130488	-3.167503112	69.758825775
H	27.721060046	-2.884119043	71.917210125
H	27.368022567	-1.178689235	71.531323492
H	26.712120067	0.219346554	73.806877210
H	29.602390737	-2.858596835	74.240802554
H	29.278636180	-1.775792686	76.547475599
H	24.691512093	-0.421986495	77.633470612
H	26.267930906	-0.990836641	78.558716924
H	26.066556791	2.571144208	81.489251844
H	26.516999186	1.431579105	68.093664255
H	22.481447076	4.181816220	68.679723825
H	21.463184528	5.233751200	74.087106727
H	23.715775764	3.700721180	84.691286444
H	21.093201364	1.217088104	79.022885277
H	26.443096990	-1.408195371	65.959923160
H	29.597055413	4.414560624	75.170293925
H	27.975589064	4.692453104	74.471607445
C	32.394500351	3.296087056	75.535676528

H	32.959395847	3.450225911	76.477015119
H	32.905447655	3.857533851	74.726996323
H	31.384217584	3.732372419	75.668218495
H	27.270340415	2.271912704	74.996853436
H	28.579655371	-1.451287090	69.324691935
H	30.264372584	-2.090299098	69.314255501

‘Substrate bound’ (C) model

C	-6.584001000	2.097609000	5.398068000
C	-4.605129000	4.070113000	2.767713000
C	-5.849988000	2.360789000	4.087567000
N	-5.427493000	3.640737000	3.887086000
O	-5.658452000	1.438722000	3.268110000
C	-3.112581000	4.161955000	3.168057000
O	-2.684518000	2.888692000	3.621633000
C	-2.283932000	4.672214000	1.977149000
O	-0.926945000	4.934412000	2.361473000
C	-2.866549000	5.975191000	1.411689000
O	-4.228788000	5.739134000	1.049398000
C	-2.082034000	6.485205000	0.207844000
O	-1.792964000	5.403239000	-0.672431000
H	-4.718499000	3.311399000	1.966070000
H	-3.018003000	4.921203000	3.987092000
H	-2.814888000	6.764657000	2.203833000
H	-7.471383000	1.470054000	5.187316000
H	-6.895438000	3.019123000	5.926066000
H	-5.587010000	4.317205000	4.638391000
H	-5.912276000	1.513410000	6.060600000
H	-1.699851000	2.931106000	3.749454000
H	-2.692551000	7.279435000	-0.283453000
H	-1.155827000	6.960155000	0.604894000
H	-1.317564000	5.762733000	-1.443790000
C	1.330686000	7.125393000	-0.989075000
C	-0.014640000	3.920211000	2.107310000
C	1.365686000	4.540759000	1.795096000
C	1.375056000	6.600231000	0.430506000
N	1.328830000	5.237245000	0.529172000
C	2.451588000	3.457487000	1.776354000
O	3.761891000	3.985478000	1.642237000
C	2.385870000	2.599191000	3.040148000
C	0.979513000	1.997132000	3.178165000
O	0.040415000	3.084106000	3.284177000
C	0.810188000	1.085812000	4.381463000
O	1.127781000	1.802896000	5.564197000
H	-0.352242000	3.290952000	1.245951000
H	2.233854000	2.765508000	0.930240000
H	2.564077000	3.245481000	3.934347000
H	0.760215000	1.390048000	2.262573000
H	1.377143000	6.336234000	-1.765120000
H	2.177761000	7.824617000	-1.138129000
H	1.294617000	4.684138000	-0.333629000
H	0.403812000	7.719431000	-1.127756000
H	4.150842000	3.753973000	0.762160000
H	-0.241171000	0.707935000	4.385273000
H	1.480329000	0.209766000	4.214391000
H	1.143231000	1.161949000	6.299089000
O	3.337929000	1.558643000	2.945311000
H	4.142594000	1.985474000	2.579431000
C	-5.052837000	5.433158000	2.178397000
H	-4.901812000	6.235947000	2.952269000
O	-6.371844000	5.365221000	1.754698000
H	-6.659744000	6.275591000	1.545647000
N	0.000000000	-2.073771000	0.000000000
C	1.318387000	-2.775906000	-0.083467000
C	1.159883000	-4.294333000	-0.027405000
C	2.255060000	-2.214182000	1.012517000
C	2.809676000	-0.888210000	0.591857000

C	4.116309000	-0.425669000	0.538282000
N	1.991686000	0.069343000	0.000000000
C	2.786360000	1.066066000	-0.423017000
N	4.071721000	0.797273000	-0.103927000
C	1.448189000	2.421658000	-4.808668000
C	1.122691000	2.617330000	-3.336422000
O	1.912898000	3.155766000	-2.527179000
O	-0.040119000	2.117246000	-2.983400000
C	7.300159000	4.061443000	-1.821875000
C	5.834195000	3.667398000	-1.607277000
N	4.972030000	3.867381000	-2.621550000
O	5.459845000	3.168633000	-0.510348000
C	-1.035864000	-3.621675000	4.125860000
C	-2.160015000	-3.937423000	3.129209000
O	-2.117442000	-3.494662000	1.953251000
C	-0.608247000	-2.151820000	3.991171000
N	-3.184491000	-4.729341000	3.554800000
C	-4.234597000	-5.198796000	2.619932000
C	-5.361984000	-4.135950000	2.478588000
O	-6.381807000	-4.173981000	3.180398000
C	-4.747578000	-6.487216000	3.288891000
C	-4.633717000	-6.186600000	4.797651000
C	-3.323797000	-5.368965000	4.891225000
N	-5.144052000	-3.190602000	1.518323000
C	-6.093211000	-2.091668000	1.271012000
C	-7.465926000	-2.619252000	0.799495000
C	-5.505657000	-1.121647000	0.216437000
C	-4.144003000	-0.560854000	0.498134000
C	-2.935670000	-0.825502000	-0.131983000
N	-3.853638000	0.391830000	1.467677000
C	-2.529979000	0.681332000	1.403031000
N	-1.943640000	-0.045229000	0.440634000
C	-9.184428000	-5.185886000	-3.225488000
C	-8.747390000	-5.292136000	-4.682804000
O	-8.019018000	-5.032584000	-2.390106000
C	-3.292335000	-5.185180000	-6.614299000
C	-3.942166000	-5.079837000	-5.267245000
C	-5.270170000	-5.329621000	-4.941909000
C	-3.302084000	-4.703232000	-4.030693000
C	-4.305188000	-4.751204000	-2.995730000
C	-1.981923000	-4.335307000	-3.692985000
N	-5.488502000	-5.135450000	-3.587479000
C	-4.000590000	-4.460582000	-1.648630000
C	-1.682697000	-4.048954000	-2.356321000
C	-2.672323000	-4.124458000	-1.337231000
C	0.440520000	-3.070734000	-6.146362000
C	1.249122000	-2.414240000	-5.053321000
C	0.664118000	-1.501547000	-4.148897000
C	2.631909000	-2.686175000	-4.928253000
C	1.441497000	-0.863748000	-3.166326000
C	3.409565000	-2.053709000	-3.944141000
C	2.817372000	-1.131108000	-3.061565000
Cu	0.000000000	0.000000000	0.000000000
O	-0.167930000	1.903071000	-0.520283000
H	0.540108000	2.540253000	-5.429683000
H	1.805388000	1.376373000	-4.930242000
H	7.585170000	4.780622000	-1.028193000
H	7.499654000	4.504127000	-2.815493000
H	5.281537000	4.269935000	-3.508000000
H	3.972594000	3.609306000	-2.537710000
H	0.210532000	-1.923570000	4.701958000
H	-1.453460000	-1.464960000	4.200144000

H	-0.246293000	-1.922783000	2.968409000
H	-1.418511000	-3.767791000	5.156315000
H	-4.083534000	-7.329844000	3.004152000
H	-5.779207000	-6.731967000	2.972421000
H	-5.494600000	-5.565418000	5.111795000
H	-4.595442000	-7.096132000	5.427679000
H	-3.358665000	-4.604570000	5.693516000
H	-2.448653000	-6.026909000	5.080872000
H	-3.772644000	-5.377005000	1.626731000
H	-9.754094000	-6.096498000	-2.932205000
H	-8.145333000	-4.408859000	-4.979338000
H	-8.145246000	-6.208998000	-4.845781000
H	-9.634996000	-5.340188000	-5.345647000
H	-8.221140000	-5.359576000	-1.493158000
H	-2.844268000	-4.217195000	-6.930239000
H	-2.463422000	-5.927060000	-6.613787000
H	-6.087411000	-5.643174000	-5.601589000
H	-1.200038000	-4.285215000	-4.462679000
H	-0.644493000	-3.782855000	-2.110035000
H	-4.773046000	-4.503138000	-0.864423000
H	-2.400484000	-3.932964000	-0.286269000
H	0.454889000	-4.178062000	-6.055884000
H	-0.616009000	-2.738104000	-6.137674000
H	-0.412596000	-1.277841000	-4.219848000
H	3.105166000	-3.400303000	-5.622583000
H	0.964462000	-0.140084000	-2.490434000
H	4.485935000	-2.276186000	-3.868146000
H	3.428758000	-0.626215000	-2.299499000
H	-0.562430000	-2.433292000	0.800472000
H	1.742078000	-2.497019000	-1.070179000
H	2.148649000	-4.785972000	-0.124190000
H	3.092686000	-2.915337000	1.189953000
H	1.697201000	-2.135934000	1.972259000
H	5.050458000	-0.869068000	0.895460000
H	2.479326000	1.924610000	-1.036470000
H	-4.174768000	-3.101122000	1.177291000
H	-6.237748000	-1.533300000	2.226152000
H	-8.164265000	-1.769871000	0.654634000
H	-6.233713000	-0.292923000	0.088885000
H	-5.450678000	-1.651533000	-0.757836000
H	-2.741807000	-1.508120000	-0.964947000
H	-4.530193000	0.845325000	2.139153000
H	-2.052490000	1.428470000	2.043067000
H	-0.139271000	2.058468000	-1.880730000
H	0.667677000	2.369729000	-0.317479000
H	4.831213000	1.494130000	-0.273386000
H	-6.410819000	-5.197892000	-3.108400000
H	-4.017931000	-5.491163000	-7.394578000
H	0.861018000	-2.834004000	-7.147113000
H	7.924786000	3.155817000	-1.685564000
H	2.245381000	3.114942000	-5.133541000
H	-9.864095000	-4.312176000	-3.088174000
H	0.706140000	-4.613279000	0.933581000
H	0.512464000	-4.669644000	-0.841876000
C	0.147128000	-4.599729000	3.919189000
H	0.944444000	-4.396545000	4.662357000
H	-0.169306000	-5.657199000	4.029656000
H	0.583731000	-4.477122000	2.907654000
H	-0.556024000	-2.315524000	-0.835224000
H	-7.372649000	-3.159313000	-0.166948000
H	-7.892575000	-3.303868000	1.554859000
H	-2.303519000	3.907188000	1.169359000

H	1.590806000	5.291904000	2.579899000
O	1.435875000	7.349855000	1.413211000

‘5-coordinate superoxide’ (D) model

N	0.000000000	-2.164283000	0.000000000
C	1.294631000	-2.850323000	0.263258000
C	1.099273000	-4.305726000	0.671011000
C	2.122553000	-2.056259000	1.301198000
C	2.734180000	-0.853059000	0.664561000
C	4.060295000	-0.511667000	0.447116000
N	1.943692000	0.076174000	0.000000000
C	2.762294000	0.948690000	-0.599733000
N	4.049106000	0.620151000	-0.346755000
C	2.622337000	0.741076000	-5.673976000
C	2.222772000	1.356557000	-4.323066000
O	3.126005000	1.681480000	-3.492865000
O	0.969732000	1.464065000	-4.120053000
C	7.975320000	3.100727000	-2.263193000
C	6.611960000	2.432338000	-2.184414000
N	5.855933000	2.415009000	-3.297391000
O	6.248559000	1.931858000	-1.091238000
C	-1.766453000	-2.448470000	3.972668000
C	-2.721916000	-3.021825000	2.922230000
O	-2.458831000	-2.955081000	1.695322000
C	-1.241665000	-1.075913000	3.528454000
N	-3.847154000	-3.636878000	3.368408000
C	-4.736238000	-4.386318000	2.451182000
C	-5.791833000	-3.448812000	1.810037000
O	-6.957398000	-3.391509000	2.228606000
C	-5.392418000	-5.419842000	3.376436000
C	-5.537686000	-4.653775000	4.702996000
C	-4.235622000	-3.839439000	4.786109000
N	-5.345491000	-2.713226000	0.755600000
C	-6.205854000	-1.716894000	0.101664000
C	-7.417132000	-2.357202000	-0.581627000
C	-5.385271000	-0.915755000	-0.929053000
C	-4.109205000	-0.366363000	-0.394801000
C	-2.797052000	-0.721359000	-0.655930000
N	-4.031081000	0.618048000	0.577961000
C	-2.723162000	0.844961000	0.867485000
N	-1.955170000	0.043512000	0.130904000
C	-8.258014000	-5.451314000	-4.159389000
C	-7.917590000	-6.482281000	-5.226449000
O	-7.518140000	-4.239912000	-4.415558000
C	-2.218564000	-7.004470000	-6.090183000
C	-3.074002000	-6.153149000	-5.200412000
C	-4.304318000	-5.577859000	-5.504305000
C	-2.781836000	-5.760431000	-3.839817000
C	-3.878687000	-4.943657000	-3.387468000
C	-1.702401000	-6.009379000	-2.962181000
N	-4.783539000	-4.851778000	-4.425235000
C	-3.900884000	-4.381125000	-2.095540000
C	-1.729424000	-5.448035000	-1.681367000
C	-2.809780000	-4.634366000	-1.251398000
C	1.508308000	-4.890823000	-5.569783000
C	0.978722000	-3.736011000	-4.747248000
C	-0.409680000	-3.491936000	-4.663502000
C	1.847928000	-2.885446000	-4.026137000
C	-0.915616000	-2.435136000	-3.887919000
C	1.347867000	-1.816725000	-3.261080000
C	-0.037680000	-1.583275000	-3.194564000
Cu	0.000000000	0.000000000	0.000000000
O	-0.268409000	1.474345000	-1.864038000

O	0.038415000	1.901590000	1.105658000
H	1.888972000	1.008517000	-6.459632000
H	2.607058000	-0.364943000	-5.565176000
H	7.992057000	3.950693000	-1.552686000
H	8.226335000	3.467972000	-3.275915000
H	6.205387000	2.867074000	-4.144025000
H	4.878100000	2.035627000	-3.330796000
H	-0.528760000	-0.677282000	4.277210000
H	-2.070295000	-0.349512000	3.414360000
H	-0.721640000	-1.134369000	2.551343000
H	-2.323075000	-2.306148000	4.919434000
H	-4.710849000	-6.287180000	3.493408000
H	-6.357705000	-5.777586000	2.973733000
H	-6.411099000	-3.976766000	4.641093000
H	-5.664444000	-5.315233000	5.581064000
H	-4.369981000	-2.868406000	5.301972000
H	-3.441023000	-4.405986000	5.315248000
H	-4.119714000	-4.837900000	1.647979000
H	-8.015654000	-5.847463000	-3.145958000
H	-8.147719000	-6.090848000	-6.238225000
H	-6.844975000	-6.758235000	-5.191918000
H	-8.512460000	-7.403021000	-5.065174000
H	-7.896962000	-3.525102000	-3.868158000
H	-1.212919000	-6.556536000	-6.244696000
H	-2.046472000	-8.012206000	-5.654065000
H	-4.884104000	-5.633908000	-6.434304000
H	-0.851107000	-6.628785000	-3.284706000
H	-0.899916000	-5.646314000	-0.987811000
H	-4.750140000	-3.774416000	-1.750268000
H	-2.787201000	-4.199248000	-0.240984000
H	2.591655000	-4.785331000	-5.774492000
H	1.361318000	-5.855695000	-5.036533000
H	-1.109365000	-4.139632000	-5.212704000
H	2.935857000	-3.055721000	-4.077292000
H	-2.004558000	-2.274587000	-3.838965000
H	2.038470000	-1.143615000	-2.730418000
H	-0.411022000	-0.719785000	-2.624699000
H	-0.715731000	-2.432672000	0.700264000
H	1.854418000	-2.819792000	-0.694112000
H	2.073979000	-4.798394000	0.861748000
H	2.926541000	-2.701730000	1.702241000
H	1.473370000	-1.783570000	2.162483000
H	4.991166000	-0.980265000	0.782427000
H	2.468949000	1.749152000	-1.285112000
H	-4.320679000	-2.662045000	0.631618000
H	-6.579790000	-1.019819000	0.886411000
H	-8.065683000	-1.568580000	-1.012780000
H	-6.032507000	-0.103606000	-1.319464000
H	-5.136828000	-1.567868000	-1.790405000
H	-2.426486000	-1.473440000	-1.357787000
H	-4.821297000	1.113678000	0.998622000
H	-2.372008000	1.588550000	1.586779000
H	0.337112000	1.514967000	-2.698874000
H	0.019172000	2.217472000	-1.280509000
H	4.901909000	1.147723000	-0.709361000
H	-5.749090000	-4.464805000	-4.371142000
H	-2.681093000	-7.142704000	-7.087030000
H	0.975110000	-4.975160000	-6.538963000
H	8.745727000	2.378506000	-1.928124000
H	3.642556000	1.044421000	-5.981258000
H	-9.347271000	-5.227464000	-4.176240000
H	0.487651000	-4.386655000	1.591435000

H	0.583957000	-4.868256000	-0.129291000
C	-0.622500000	-3.450141000	4.240876000
H	0.080509000	-3.024942000	4.984999000
H	-0.979310000	-4.426599000	4.633169000
H	-0.052719000	-3.649416000	3.311735000
H	-0.357158000	-2.480600000	-0.909414000
H	-7.094905000	-3.027334000	-1.407092000
H	-8.007780000	-2.944329000	0.143530000
O	0.302623000	2.967257000	0.414633000

‘4-coordinate superoxide'- (E) model

N	-0.192703000	-2.156039000	0.000000000
C	1.042463000	-2.967297000	0.163259000
C	0.752399000	-4.374718000	0.660888000
C	2.030303000	-2.253640000	1.112717000
C	2.668587000	-1.072880000	0.465886000
C	3.985913000	-0.855371000	0.098298000
N	1.917445000	0.000000000	0.000000000
C	2.753266000	0.837783000	-0.636031000
N	4.009560000	0.341903000	-0.590243000
C	2.800136000	1.226446000	-5.038406000
C	2.489930000	1.950270000	-3.708904000
O	3.477833000	2.462652000	-3.073850000
O	1.298383000	1.973591000	-3.300235000
C	8.116340000	2.909934000	-1.196594000
C	6.809539000	2.305631000	-1.672558000
N	6.098131000	2.976972000	-2.590907000
O	6.431824000	1.210952000	-1.181188000
C	-2.102942000	-2.724590000	4.002289000
C	-3.054371000	-3.159567000	2.879482000
O	-2.753908000	-2.981571000	1.672259000
C	-1.450334000	-1.382282000	3.637103000
N	-4.231341000	-3.748709000	3.228422000
C	-5.136314000	-4.311328000	2.199815000
C	-6.078356000	-3.218727000	1.629322000
O	-7.226929000	-3.053218000	2.064101000
C	-5.918553000	-5.384371000	2.973062000
C	-6.059534000	-4.782007000	4.383726000
C	-4.701120000	-4.076416000	4.599267000
N	-5.554030000	-2.484168000	0.607284000
C	-6.297983000	-1.373605000	-0.008777000
C	-7.592323000	-1.835720000	-0.686160000
C	-5.405960000	-0.658106000	-1.041021000
C	-4.103437000	-0.174516000	-0.506134000
C	-2.805187000	-0.539488000	-0.814171000
N	-3.984455000	0.792572000	0.480254000
C	-2.668716000	1.002898000	0.735458000
N	-1.929082000	0.208642000	-0.043007000
C	-8.485703000	-4.822542000	-4.408286000
C	-8.104279000	-5.409141000	-5.766510000
O	-7.625995000	-3.746156000	-3.984133000
C	-2.407696000	-6.150987000	-6.471034000
C	-3.229290000	-5.443384000	-5.442959000
C	-4.537351000	-4.982794000	-5.545654000
C	-2.805257000	-5.129086000	-4.102854000
C	-3.913598000	-4.494305000	-3.438943000
C	-1.590920000	-5.309510000	-3.411071000
N	-4.947677000	-4.417304000	-4.348087000
C	-3.828202000	-4.079532000	-2.094837000
C	-1.500424000	-4.866001000	-2.090638000
C	-2.606812000	-4.270127000	-1.429070000
C	1.396589000	-4.314281000	-5.587153000
C	0.990701000	-3.150481000	-4.715103000
C	-0.331321000	-2.661214000	-4.704372000
C	1.941260000	-2.524278000	-3.876698000
C	-0.694988000	-1.573163000	-3.889452000
C	1.587804000	-1.429429000	-3.076317000
C	0.267417000	-0.944555000	-3.083735000
Cu	0.000000000	0.000000000	0.000000000
O	-0.012421000	2.201897000	0.310333000

H	1.879242000	1.062160000	-5.629211000
H	3.237708000	0.232645000	-4.806666000
H	7.999735000	3.201235000	-0.132519000
H	8.431014000	3.795825000	-1.779516000
H	6.438105000	3.880048000	-2.925834000
H	5.130687000	2.665367000	-2.885834000
H	-0.807959000	-1.029145000	4.468368000
H	-2.210399000	-0.603596000	3.426627000
H	-0.817502000	-1.480527000	2.732601000
H	-2.699743000	-2.579427000	4.925226000
H	-5.317467000	-6.316160000	3.002196000
H	-6.891216000	-5.607424000	2.497037000
H	-6.880784000	-4.040558000	4.387579000
H	-6.255960000	-5.538367000	5.166874000
H	-4.789145000	-3.159471000	5.215056000
H	-3.971716000	-4.752606000	5.092162000
H	-4.522401000	-4.722308000	1.373278000
H	-8.405885000	-5.595510000	-3.616657000
H	-8.094469000	-4.624903000	-6.552455000
H	-7.109095000	-5.892161000	-5.734480000
H	-8.843743000	-6.180241000	-6.065982000
H	-7.827564000	-2.960764000	-4.531827000
H	-1.482791000	-5.587266000	-6.724593000
H	-2.075770000	-7.149552000	-6.113136000
H	-5.218078000	-5.017249000	-6.404154000
H	-0.725975000	-5.772361000	-3.909011000
H	-0.543951000	-4.983794000	-1.567441000
H	-4.691059000	-3.640512000	-1.573561000
H	-2.519021000	-3.964992000	-0.374328000
H	1.677295000	-5.196658000	-4.972622000
H	0.573409000	-4.623474000	-6.260316000
H	-1.090519000	-3.140816000	-5.340211000
H	2.980184000	-2.892843000	-3.866680000
H	-1.733193000	-1.206058000	-3.905504000
H	2.346142000	-0.932938000	-2.456389000
H	0.021328000	-0.041917000	-2.501369000
H	-0.906121000	-2.400066000	0.708960000
H	1.510059000	-3.013217000	-0.841534000
H	1.660128000	-5.008683000	0.598167000
H	2.821474000	-2.965808000	1.416828000
H	1.484770000	-1.965800000	2.038611000
H	4.890585000	-1.446243000	0.272140000
H	2.481810000	1.743564000	-1.192344000
H	-4.525476000	-2.521822000	0.510675000
H	-6.568054000	-0.652119000	0.797263000
H	-8.142729000	-0.952242000	-1.067503000
H	-5.989500000	0.191208000	-1.453762000
H	-5.197095000	-1.341217000	-1.889026000
H	-2.464011000	-1.282502000	-1.541026000
H	-4.756380000	1.289085000	0.933138000
H	-2.291515000	1.723843000	1.464956000
H	4.898444000	0.778802000	-0.984123000
H	-5.920158000	-4.087099000	-4.159367000
H	-2.972557000	-6.301736000	-7.412288000
H	2.278562000	-4.066153000	-6.212839000
H	8.909138000	2.137508000	-1.237807000
H	3.546693000	1.788043000	-5.636603000
H	-9.539928000	-4.468339000	-4.417832000
H	0.434629000	-4.352461000	1.724184000
H	-0.053912000	-4.873288000	0.096587000
C	-1.039404000	-3.814153000	4.289853000
H	-0.380224000	-3.487253000	5.118163000

H	-1.503120000	-4.779907000	4.573883000
H	-0.411842000	-3.986784000	3.393368000
H	-0.614206000	-2.390038000	-0.905698000
H	-7.385213000	-2.507414000	-1.544982000
H	-8.238578000	-2.364405000	0.036863000
O	0.954842000	2.788866000	0.906670000

'3-coordinate Hisbrace' (F) model

N	-0.172133000	-2.058504000	0.164332000
C	1.114874000	-2.766577000	0.408919000
C	0.853705000	-4.205021000	0.826930000
C	1.962461000	-2.012558000	1.457671000
C	2.604151000	-0.779788000	0.908931000
C	3.929744000	-0.377750000	0.873471000
N	1.861697000	0.184636000	0.234327000
C	2.710795000	1.132614000	-0.197221000
N	3.965756000	0.817199000	0.179790000
C	2.901442000	1.396143000	-4.872364000
C	2.549386000	2.005970000	-3.508117000
O	3.496084000	2.432345000	-2.765966000
O	1.334509000	2.042524000	-3.153062000
C	8.217646000	3.079631000	-1.030552000
C	6.783805000	2.605867000	-1.164528000
N	6.228020000	2.625412000	-2.386718000
O	6.177244000	2.221600000	-0.131879000
C	-2.007563000	-2.554229000	4.188707000
C	-2.953948000	-2.976396000	3.056951000
O	-2.647522000	-2.788081000	1.850052000
C	-1.371357000	-1.196346000	3.855584000
N	-4.131272000	-3.565745000	3.397444000
C	-5.038303000	-4.116555000	2.364805000
C	-5.975240000	-3.015733000	1.802097000
O	-7.124186000	-2.857451000	2.236935000
C	-5.824292000	-5.194151000	3.128970000
C	-5.958228000	-4.612310000	4.549768000
C	-4.598427000	-3.908592000	4.767315000
N	-5.455292000	-2.264104000	0.788402000
C	-6.229670000	-1.165576000	0.186517000
C	-7.491017000	-1.666023000	-0.520118000
C	-5.380575000	-0.392468000	-0.838890000
C	-4.054238000	0.064336000	-0.351856000
C	-2.786043000	-0.304916000	-0.755065000
N	-3.861947000	0.984748000	0.667013000
C	-2.532590000	1.162921000	0.858269000
N	-1.855588000	0.393113000	-0.001372000
C	-8.041537000	-4.710760000	-4.168611000
C	-8.002973000	-5.239444000	-5.600468000
O	-7.401006000	-3.429062000	-4.009576000
C	-2.306390000	-5.981290000	-6.304992000
C	-3.088473000	-5.198629000	-5.300839000
C	-4.296202000	-4.516523000	-5.505542000
C	-2.767352000	-5.014290000	-3.907694000
C	-3.815782000	-4.215991000	-3.331183000
C	-1.711652000	-5.467852000	-3.086460000
N	-4.723727000	-3.928165000	-4.339677000
C	-3.814782000	-3.859170000	-1.973306000
C	-1.721719000	-5.126200000	-1.728672000
C	-2.748797000	-4.320934000	-1.175162000
C	1.497895000	-4.144584000	-5.421111000
C	1.050217000	-3.023422000	-4.513052000
C	-0.297810000	-2.611578000	-4.458561000
C	1.990189000	-2.337819000	-3.707985000
C	-0.691046000	-1.521837000	-3.660519000
C	1.605189000	-1.247122000	-2.915651000
C	0.263128000	-0.823502000	-2.903023000
Cu	0.000000000	0.000000000	0.000000000
H	2.009520000	1.323688000	-5.523489000

H	3.293887000	0.370500000	-4.709766000
H	8.234134000	3.969610000	-0.369345000
H	8.686806000	3.337210000	-1.998172000
H	6.771488000	2.979492000	-3.175527000
H	5.209079000	2.422952000	-2.549946000
H	-0.700599000	-0.872680000	4.675748000
H	-2.142130000	-0.414935000	3.702911000
H	-0.770949000	-1.254874000	2.926316000
H	-2.606362000	-2.435024000	5.114307000
H	-5.227648000	-6.128587000	3.142314000
H	-6.798971000	-5.406511000	2.652801000
H	-6.779157000	-3.870322000	4.568513000
H	-6.148886000	-5.380344000	5.323015000
H	-4.684282000	-2.997429000	5.391388000
H	-3.864498000	-4.588400000	5.248649000
H	-4.426998000	-4.528837000	1.537876000
H	-7.498809000	-5.393897000	-3.484211000
H	-8.530498000	-4.551944000	-6.294570000
H	-6.962044000	-5.367536000	-5.957230000
H	-8.508498000	-6.225173000	-5.653410000
H	-7.862330000	-2.779256000	-4.577837000
H	-1.308914000	-5.525980000	-6.490494000
H	-2.116463000	-7.016799000	-5.951594000
H	-4.884907000	-4.423237000	-6.426572000
H	-0.902347000	-6.086768000	-3.503129000
H	-0.926136000	-5.512047000	-1.077381000
H	-4.630587000	-3.266151000	-1.535431000
H	-2.726304000	-4.061095000	-0.104408000
H	1.914623000	-4.993651000	-4.838639000
H	0.659770000	-4.532020000	-6.032165000
H	-1.051702000	-3.138658000	-5.062541000
H	3.046873000	-2.650335000	-3.725484000
H	-1.744132000	-1.199929000	-3.666960000
H	2.355698000	-0.702899000	-2.324846000
H	-0.009758000	0.099884000	-2.364671000
H	-0.878436000	-2.266688000	0.898221000
H	1.661575000	-2.751519000	-0.555723000
H	1.805345000	-4.756618000	0.958281000
H	2.756793000	-2.688161000	1.827185000
H	1.320042000	-1.776856000	2.335740000
H	4.832252000	-0.841611000	1.283092000
H	2.466649000	1.982266000	-0.846219000
H	-4.427990000	-2.289226000	0.685248000
H	-6.534946000	-0.472494000	1.003880000
H	-8.077654000	-0.799790000	-0.886197000
H	-5.984068000	0.473031000	-1.182351000
H	-5.208792000	-1.027798000	-1.730986000
H	-2.493940000	-1.014244000	-1.534056000
H	-4.598619000	1.471233000	1.185418000
H	-2.098153000	1.837548000	1.601674000
H	4.839264000	1.407819000	-0.024634000
H	-5.690699000	-3.543417000	-4.208377000
H	-2.833784000	-6.039094000	-7.276466000
H	2.298792000	-3.806274000	-6.111100000
H	8.810377000	2.288905000	-0.529113000
H	3.696345000	1.987368000	-5.369614000
H	-9.091522000	-4.645977000	-3.805378000
H	0.297191000	-4.241062000	1.784670000
H	0.255189000	-4.734574000	0.065431000
C	-0.938098000	-3.644456000	4.455895000
H	-0.272011000	-3.326361000	5.282035000
H	-1.400518000	-4.612393000	4.732983000

H	-0.314492000	-3.809817000	3.555142000
H	-0.581619000	-2.386369000	-0.719957000
H	-7.238460000	-2.300873000	-1.395257000
H	-8.124063000	-2.246968000	0.174773000

'Superoxide' (G) model

O	-0.526979000	0.826667000	0.000000000
O	0.833646000	0.826667000	0.000000000
